# Physiological response to self-compassion versus relaxation in a clinical population

**DOI:** 10.1371/journal.pone.0272198

**Published:** 2023-02-07

**Authors:** Iona Naismith, Clara Sophie Otto Scheiber, Daniela Gonzalez Rodriguez, Nicola Petrocchi

**Affiliations:** 1 Department of Psychology, University of the Andes, Bogota, Colombia; 2 Department of Economics and Social Sciences, John Cabot University, Rome, Italy; Friedrich-Alexander-University Erlangen-Nurnberg, GERMANY

## Abstract

**Background:**

Compassion-focused imagery (CFI) can be an effective emotion-regulation technique but can create threat-focused responses in some individuals. However, these findings have been based on tasks involving receiving compassion from others.

**Aims:**

This study sought to compare responses CFI involving *self*-compassion to relaxation and a control task, and to see whether any threat-responses to self-compassion and relaxation decrease with practice.

**Method:**

25 participants with depression/anxiety symptoms and high self-criticism and/or low self-compassion engaged in three tasks (control task, relaxation imagery, and CFI) at three or four separate testing sessions, every three days. Heart-rate variability (HRV) was used to explore group-level differences between tasks. Additionally, we identified how many individuals showed a clinically significant change in HRV in response to compassion (compared to baseline) and how many showed such a change during relaxation (compared to baseline).

**Results:**

During session 1, more individuals had a clinically significant increase in HRV in response to CFI (56%) than in response to relaxation (44%), and fewer had a clinically significant decrease in HRV during CFI (16%) than during relaxation (28%). Comparing the group as a whole, no significant differences between tasks were seen. Repeated sessions led to fewer positive responses to CFI, perhaps reflecting habituation/boredom.

**Conclusions:**

*These preliminary findings suggest that* in high self-critics (those most likely to find self-compassion difficult), self-compassionate imagery is no more challenging than standard relaxation tasks. For both compassion and relaxation, some individuals respond positively and others negatively. For those who are not benefiting, practice alone is *not* sufficient to improve response. Effects may differ for other compassion tasks.

**Trial registration:**

Trial number: NCT04647318.

## Introduction

Self-criticism represents a crucial phenomenon in a variety of mental disorders [1]. It typically takes the form of invalidating or openly aggressive self-talk, which stimulates the same neurophysiological systems as criticism generated by others [2]. As a result, highly self-critical individuals show an over-stimulated and poorly regulated threat emotional system, with subsequent negative affectivity and underdeveloped capacities for emotional regulation [3, 4]. Self-criticism shows positive associations with symptoms of depression, eating disorders, social anxiety disorder, and personality disorders as well as psychotic symptoms and interpersonal problems [1]. Whilst most studies on these relationships are correlational, longitudinal studies support the idea that self-criticism plays a causal role in depression and anxiety [1].

Over the past 20 years, several interventions aiming at increasing compassion and self-compassion as an antidote to self-criticism have been developed [5]. One of the most empirically validated is Compassion Focused Therapy (CFT [6]). CFT has proposed a model of affect regulation involving three evolved emotional systems: the threat and self-protection system, the drive and resource-seeking system, and the soothing system. Some emotional difficulties such as high shame and self-criticism can be conceptualized as stemming from a threat system that has been defensively hyperactivated by interpersonal traumas such as abuse, bullying, or high expressed emotion in the family of origin [7, 8]. CFT conceptualizes another cause of emotional difficulties as an under-activated soothing system. Evidence suggests that parental warmth and affection activates the soothing system to enable infants to emotionally regulate threat-focused emotions such as fear, anger and disgust [9, 10]. Adults who have had positive caregiving experiences emotionally regulate by activating memories, emotions or schemas of support, encouragement and validation. In contrast, a lack of parental warmth and affection in infancy is associated with difficulties with self-soothing [11].

The primary aim of CFT is to increase acceptance and compassion for one’s own suffering in order to generate a self-soothing response [12]. Self-soothing operates through the stimulation of particular types of positive affect (contentment, safeness, lovability, serenity; [13]), and through increased activity of the vagus nerve and corresponding higher heart rate variability [14, 15]. A recent meta-analysis suggested that compassion-based interventions hold promise as a form of intervention to reduce depression, anxiety, and psychological distress and increase well-being [5].

### Compassion focused techniques

Compassion-focused imagery (CFI) is a key technique in CFT, which involves visualizing compassion towards others, or imagining people, places or objects directing compassion towards oneself [16]. The technique is supported by evidence that mental images can evoke greater emotional responses than verbal representations [17]. CFI aims to activate affiliative positive affect and to create long-lasting change by helping the client practice their ‘compassionate self´ and integrate this into their sense of self-identity, so that it shapes future patterns of thinking, behaving, feeling and attention [6]. Single trials of CFI in non- or sub-clinical populations have shown a reduction of negative affect, an increase in self-esteem, and physiological changes associated with the attenuation of threat-defensive behaviors [18–20]. Regular CFI practice has increased self-compassion and reduced negative affect in clinical and non-clinical populations [20–23].

Other studies have found that compassion-focused interventions impact positive affect more than negative affect [24]. Such findings reflect theories arguing that distinct physiological processes underlie feelings of threat and safeness, and that compassion focused practices work directly on activating soothing positive affect [6]. As discussed by Petrocchi et al., [19]: “Differently from positive reappraisal and cognitive restructuring, which are mostly aimed to ‘restructure’ and change the very content of the self-critical process (e.g. ‘I’m not so bad; I don’t have enough evidence supporting this negative assumption about myself’), thus improving negative affect, compassion-focused coping may provide individuals with an effective way to accept negative emotions, which might not be directly impacted by the intervention” (p. 534).

Despite these promising findings, negative reactions to CFI have been observed. Some individuals have shown increased stress hormone release during CFI [25], or decreased HRV indicating a threat-focused response [20]. Thus, the assessment and treatment of factors that inhibit a positive response to CFI is important. Qualitative research with clinical populations has identified multiple inhibitors of compassion, particularly in clients with history of abuse or neglect [26–29]. Different inhibitors may arise depending on which ‘flow’ of compassion we experience: self-compassion, compassion from others, or compassion towards others [30, 31]. For example, individuals who fear being reliant on others will have difficulties receiving compassion from others; whilst self-compassion may be inhibited by feeling undeserving or believing that self-compassion will prevent attainment of high standards.

To date, almost all studies of CFI have involved imagining receiving compassion from *others* [20, 23, 25]. To our knowledge two exceptions exist, one which compared self.compassion to relaxation imagery and found no significant differences, and another which found that self-compassion was more soothing than self-critical imagery; however, both used non-clinical populations [32, 33]. Thus, there is a need to explore the acceptability of imagery involving *self*-compassion in clinical subjects. In fact, research has shown that, among the three orientations of compassion, self-compassion shows the highest correlations with depression, anxiety, stress and well-being [31]. Moreover, whilst negative reactions to self-compassion interventions have been theorized and anecdotally reported in the self-compassion literature [34], and examined in relation to basic values [35], so far no experimental study has investigated the acceptability of *self-*compassionate imagery in a clinical population.

Whilst theories of compassion inhibitors theorize that these threat-responses are towards compassion or affiliative relating specifically, there is also evidence to suggest that negative responses to CFI may reflect a response to something distinct from compassion, at least in some individuals. For example, Duarte and colleagues [25] found that high self-critics released stress hormones when trialing CFI, but also when they imagined relaxing scenery. This could reflect a conditioned fear response to positive affect in general, not simply affiliative emotions generated by CFI. It could also reflect difficulties observing and tolerating emotions and bodily sensations, since relaxing and compassionate imagery exercises typically involve mindful attention on the body, breathing and feelings. Thus, identifying the cause of any threat-response to CFI requires disentangling these factors.

Furthermore, whilst some studies have explored effects of repeated practice, they are limited by reporting only group level effects [21–23]. Repeated exposure to compassion in a safe context should be successful if threat responses represent task unfamiliarity or conditioned fear towards compassion, for example due to experiencing both care and maltreatment from the same person [6], but this requires further empirical exploration in clinical populations.

Another limitation of current literature is its reliance on self-report data, since emotional self-awareness can be limited in clinical populations [36]. An alternative is to use physiological measures like heart rate variability (HRV), which represents the variation in the times between each heartbeat and measures the dynamic balancing of the sympathetic and parasympathetic nervous systems. Low HRV indicates a chronic state of perceived high-threat due to predominantly sympathetic activity, whilst high HRV indicates the simultaneous activity of parasympathetic and sympathetic systems, and the adaptive ability to downregulate psychophysiological arousal using the vagal nerve [15, 37, 38]. Compassion interventions aim to increase parasympathetic activity and decrease sympathetic activity though giving/receiving care, and therefore HRV offers a useful form of measurement to assess the effectiveness of compassion focused interventions [39]. Engaging in a 5-minute mindfulness or compassion meditation is associated with increases in both HRV and positive affect, yet prior studies have found these two outcomes to not show strong correlations with one another [40, 41]. This has been found in other studies and has been hypothesized to reflect low validity of self-report measures due to social desirability bias or lack of emotional self-awareness [42].

The present study explores two empirical questions. Firstly, how clinical participants initially respond to *self*-compassionate imagery, and specifically, whether there is evidence of threat-focused responses. Secondly, whether 3–4 repeated trials of self-compassionate imagery are sufficient to reduce any threat response that may arise. We aimed to compare compassion to relaxation, to control for elements common to both tasks (e.g. mental imagery, diaphragmatic breathing), given that Duarte et al. [25] found that both relaxation and compassionate imagery produced a threat-like response for some individuals. We also compared this to a control task of reading a magazine.

## Methods

### Participants

Twenty-five participants (20 women) aged 18 to 35 (*M* = 24.8, *SD* = 5.2) living in Bogota, Colombia participated in the study. They were recruited as part of a separate study offering psychotherapy to individuals with clinical anxiety and/or depression. Therapy was begun at the end of the present study. Participants were recruited via the social media page of a private university, and by contacting individuals on a waitlist for a clinic offering affordable psychotherapy to low and middle-income people.

Inclusion criteria were (i) being ≥18 years old, (ii) a clinical level of depression or anxiety on the Overall Anxiety Severity and Impairment Scale [43] or Overall Depression Severity and Impairment Scale [44], and (iii) high self-criticism or low self-reassurance, as measured by the Forms of Self-Criticism/Attacking and Self-Reassuring Scale [45]. Specific cut-offs are outlined in the Measures section. Inclusion criteria (ii) and (iii) were chosen so that the sample was representative of individuals who are typically offered CFT. We did not constrict the sample to only one diagnosis since self-criticism/low self-compassion are transdiagnostic problems. Exclusion criteria were screening positively for bipolar, personality disorder, or substance dependence on the Mood Disorder Questionnaire [46], Standardised Assessment of Personality Abbreviated Scale [47] or Severity of Dependence Scale [48] (since compassion-focused imagery without prior groundwork is contraindicated or not evidence-based for these populations), or screening positively for significant suicidal intent (since this risk could not be managed in the study). In total, 76 individuals completed an initial screening questionnaire. Twenty-five met study criteria and entered the study as participants.

Since no study to date has explored the exact hypotheses of this study, the target sample size was set at N = 25 based upon other studies exploring effects of CFI on physiology, which have total samples ranging from 22 to 25 participants [20, 25] and found significant differences in physiological measures during CFI versus control tasks.

### Measures

All measures were administered in Spanish. For measures that had not been validated in Spanish (state affect, SAPAS), measures were translated using the standard forward-and-back translation method, with two bilingual speakers, one native British and one native Colombian (authors 1 and 3).

#### Assessment measures

*Heart rate variability*. The physiological measurement system BIOPAC MP150 [49] was used to collect ECG data as the primary outcome. Specifically, the electrocardiogram amplifier module (ECG100C) was connected to the BIOPAC system to record ECG signals during each task. A standard electrode configuration (right clavicle and precordial site V6) was used for collecting ECG data and electrode gel was applied to increase the conductivity between skin and electrode. ECG signals were displayed on a laptop, using AcqKnowledge v. 4.1 [49]. The ECG signal was digitized at 2000 Hz and inspected offline using Kubios software [50]. Successive R waves (identified by an automatic beat detection algorithm) were visually inspected, and any irregularities were edited. A time domain index of HRV, the Root mean square of successive differences between heartbeats (RMSSD) was then obtained for baseline, induction and each experimental condition using HRV Analysis Software [51]. RMSSD is obtained by calculating each successive time difference between heartbeats in ms, squaring each value, and then finding the square root of the average of the total. RMSSD was chosen because, according to the Task Force guidelines [52], it reflects the integrity of vagus nerve-mediated autonomic control of the heart. Additionally, unlike high frequency (HF) measures, a cut-off for clinically-significant change has been proposed (see Analysis section).

**State affect** was measured immediately before and after the compassion-focused imagery as a secondary outcome. Positive affect was measured with two items from the Relaxed positive affect scale (calm, relaxed) by Gilbert and colleagues [13] and with two items from their Safe/warmth positive affect scale (safe, content). These were combined into one *soothing* positive affect scale as it produced excellent internal consistency (α = 0.91). Negative affect was measured with four items selected to tap into threat-focused emotions (anxious, distressed, vulnerable, insecure) and showed excellent internal consistency (α = 0.90). Participants rated on a 10-point scale to what extent they were experiencing each feeling at that moment, from 1 =“none” to 10 =“extremely”, producing total scores from 4–40 for negative affect and positive affect.

#### Screening measures

**Overall Anxiety Severity and Impairment Scale (OASIS; [43])** is a 5-item self-report scale that evaluates anxiety frequency, intensity, behavioral avoidance, and functional impairment. Items are scored on a five-point Likert scale from 0–4, producing a total score from 0 to 20, with higher scores indicating greater anxiety. A score of ≥8 is indicative of clinically significant symptoms of anxiety. For this study the Colombian validation was used, which produced excellent internal consistency (α = .92) [44].

**Overall Depression Severity and Impairment Scale (ODSIS; [45])** consists of five items equivalent to those of the OASIS but assessing effects of depression. Items are also scored from 0–4. A score of ≥8 is indicative of clinically significant symptoms of r depression. This study used the Colombian validation, which produced high internal consistency (α = .89) [44]).

### Forms of Self-Criticism/Attacking and Self-Reassuring Scale (FSCRS, [53])

The FSCRS consists of 22 items rated on 5-point Likert scales, making up three components: self-inadequacy; self-hatred; and self-reassurance (ability to reassure oneself during difficulties). Factor analysis indicates that a three-factor model best fits the data; although self-inadequacy and self-hatred correlate highly [53]). The present study used a validated Spanish translation of the scale [54] which found moderate to high internal consistency for all subscales (α = .71 to α = .88). Participants were required to score ≥24 on self-inadequacy, ≥ 8 on self-hatred, or ≤18 on self-reassurance. These cut-offs correspond to 0.5 SD above the mean in self-inadequacy/self-hatred, and 0.5 SD below the mean on self-reassurance, based on a validation of the FSCRS in Colombia [55].

**Mood Disorder Questionnaire** (MDQ, [46]) is a self-report screen for bipolar disorder, with a sensitivity of 0.73 and a specificity of 0.90 for identifying a diagnosis of bipolar [46]. Question 1 contains 13 dichotomous items asking about lifetime prevalence of behaviours such as being overly talkative or excessive spending. Question 2 asks whether several of these have occurred simultaneously. Question 3 asks about how problematic such behaviors were. A positive screen is characterized by responding “Yes” to 7+ items in question 1, “yes” to question 2, “Moderate” or “Serious” to question 3. This study used the validated Spanish version [56].

**Standardised Assessment of Personality–Abbreviated Scale (SAPAS; [47])** is a self-report screen for diagnosis of personality disorder, consisting of eight yes/no items covering topics such as impulsivity, difficulties making friends, trusting others, losing one´s temper. A cut-off score of 4 was selected following recent recommendations, which has good sensitivity (0.83) and specificity (0.80) for classifying a diagnosis of personality disorder [57].

**Severity of Dependence Scale (SDS; [48])** is a 5-item self-report screen for substance use. Items enquire about respondents’ perception of control and concern regarding substance use over the last 12 months, and are rated from 0 (never/almost never) to 3 (always/almost always). A cut-off score of 3 was selected for alcohol, which produces good sensitivity (72%) and specificity (86%) [58] and 5 for other substances, following standard guidelines [48]. The Spanish validation was used for this study [59].

*Suicidality*. Participants completed a self-report screen with the following items: “On a scale of 1 to 7, what is your intent to end your life right now?” from 1 (low) to 7 (high), and “Are you uncertain about being able to control suicidal impulses?” (Yes/No). Participants were excluded for responding ≥5 on item 1 or “yes” to item 2 [60].

### Procedure

All procedures were performed in accordance with the Ethical Principles of Psychologists and Code of Conduct as set out by the BABCP and BPS. Informed consent was obtained from all individual participants included in the study. Required ethical approval was obtained from the University of the Andes ethics committee.

Recruitment and data collection were carried out between September 2018 and October 2019. Participants completed an initial screening questionnaire online that consisted of reading the study information and completing informed consent, demographics questions, and all questionnaires described in measures. Non-eligible participants were given details for other psychological services.

**Fig 1:**
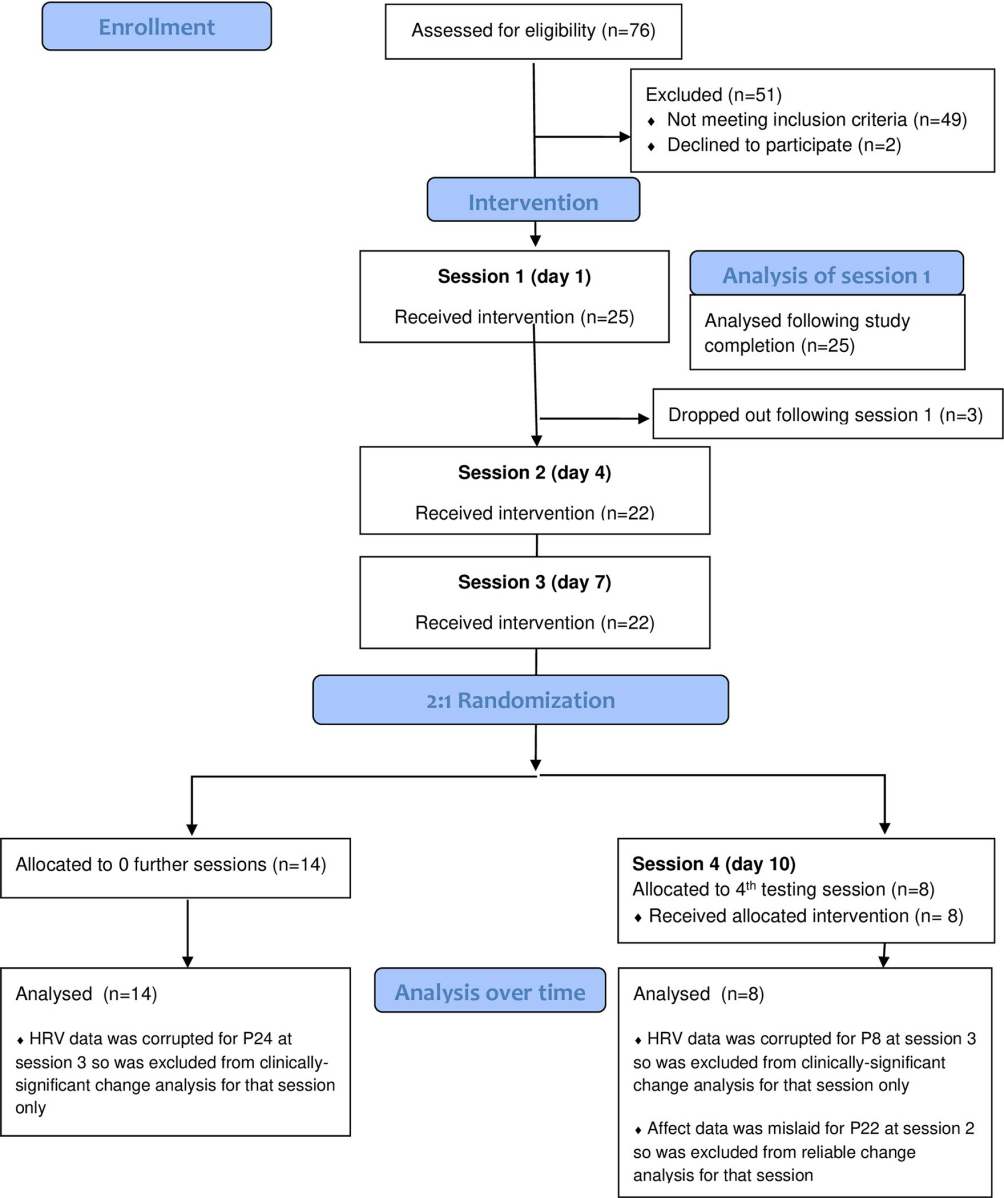
CONSORT diagram.

Eligible participants were invited to attend individual in-person sessions on university premises, in which physiological and self-report responses were collected. Participant flow is presented in Fig 1. The three participants who dropped out cited external factors such as work/caring commitments or physical illness. HRV data was lost for two participants at session 3 due to file corruptions and affect data for one participant at session 2 was mislaid (see Fig 1). At session 3, participants were randomly assigned to attend none or one additional session (i.e. 3–4 sessions in total). Collecting four datapoints offers more information but was predicted to increase dropout prior to clients receiving the subsequent therapy for a related trial. Therefore, a randomization sequence was drawn up in Excel 2016 by Author 1 prior to the study starting, with a 2:1 ratio for 3 or 4 sessions respectively. Randomization was conducted at session 3 so that prior dropout did not unbalance the allocation. Author 1 (who did not have access to participant data at the time) placed participants into the randomization sequence chronologically, based on study entry date/time. Of the 22 participants who attended session 3, eight participants were assigned to four baselines (see Fig 1). Those assigned to three baselines were not assessed further. Missing data is outlined in Fig 1.

**Fig 2:**
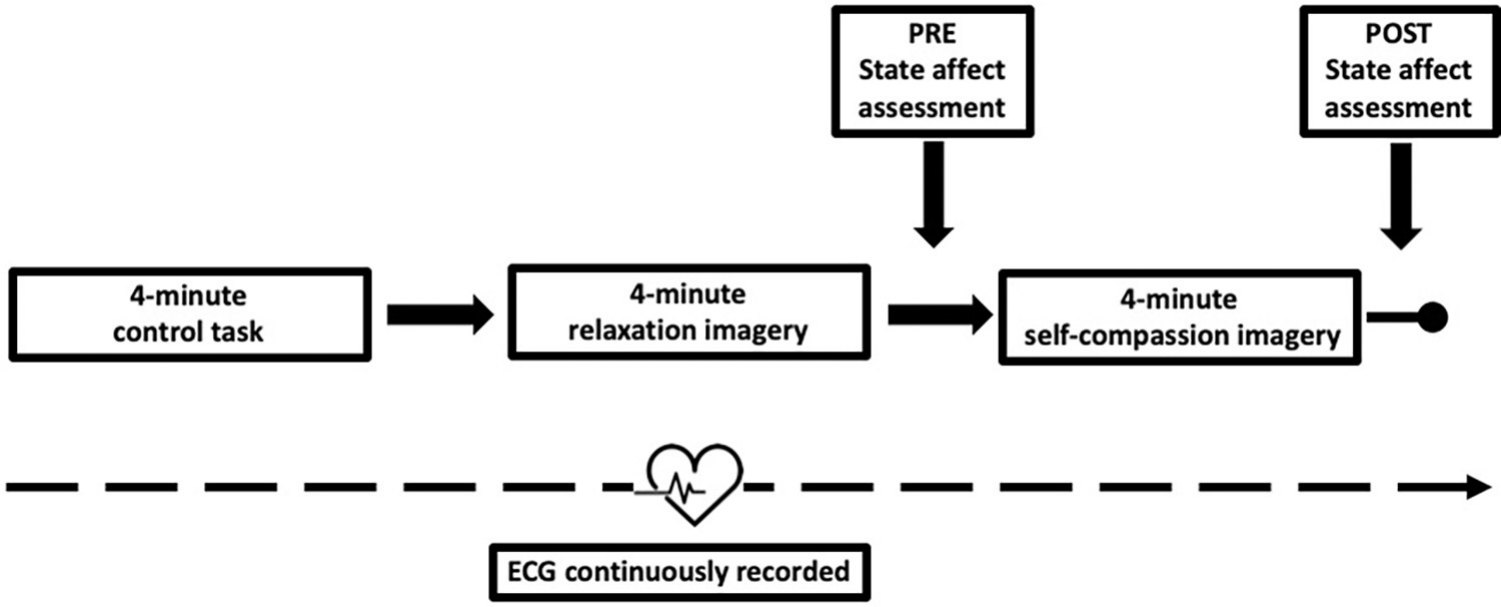
Outline of one experimental session.

Participants were asked to avoid exercising, drinking alcohol/caffeine, smoking nicotine and eating during the two hours prior to each testing session, since these are possible confounds to HRV measurements. We also confirmed each session that participants had not made any medication changes. HRV was measured by a research assistant whilst the participant engaged in three 4-minute activities, always in the following order: (i) reading a local city culture magazine (control task), (ii) engaging in relaxation imagery involving walking through a forest or on a beach, and (iii) engaging in self-compassion imagery. The order remained constant to prevent the effects of CFI from contaminating the relaxation task. An outline of one experimental session is illustrated in Fig 2.

The 4-minute relaxation imagery involved the following components (i) closing one´s eyes, breathing deeply and bringing mindful awareness to the breath, (ii) relaxing the body and releasing tension, (iii) multisensory mental imagery of a beach or a forest (this alternated each session), for example, seeing sparkling water, feeling warm sand underfoot, hearing waves and smelling salty air, before settling down on a chair to further absorb the scene, (iv) noticing feelings of relaxation that may arise.

The 4-minute self-compassion imagery script involved the following components: (i) informing participants that showing ourselves self-compassion is an effective emotional-regulation tool, and that it does not matter whether or not we believe ourselves to have the characteristics for being compassionate, the important thing is to imagine that we have them; (ii) closing one´s eyes, putting a hand on the heart, breathing deeply and attending mindfully to the breath, (iii) imagining oneself embodying the four characteristics of compassion according to Gilbert [6]: wisdom (knowing that making mistakes is human nature, that life is hard, and that kind encouragement is more effective than criticism), strength (to tolerate emotions and accept mistakes), warmth (e.g. tone of voice, facial expression), and commitment to alleviate and prevent suffering; (iv) imagine wishing oneself happy, at peace, and free of suffering using a warm voice tone, (v) noticing the feelings that arise. The original script is provided in S1 Text. Audio-recordings of scripts were used for both imagery exercises to increase homogeneity across applications.

Research assistants were not blinded to task type (as they were present in the room to collect HRV data) or to session number (as only two research assistants collected all data so were aware of how many sessions participants had already attended); however, all data was collected via self-report or physiological measures. The trial was registered retrospectively at clinicaltrials.gov (NCT04647318).

### Data analysis

Data are publicly available through Open Science Framework (https://osf.io/jpgzm/?view_only=a9acf35e1f1246bdb9a5b22b3c091050). The analyses were carried out using SPSS Version 24 [61]. An alpha level of .05 was used for all statistical tests, except where highlighted.

First, a one-way ANOVA was conducted to estimate differences in RMSSD during the self-compassion imagery, relaxation imagery and control task at session 1. Paired sample *t*-tests were also used to test changes in self-reported affect from pre-CFI to post-CFI (as noted in Methods, affect was not measured during the two other tasks to avoid respondent fatigue). Cohen´s d was used to report effect sizes.

Group-level analyses can often mask important clinical information: for example, they do not distinguish a situation where some participants show strong threat-responses and others show strong positive responses from a situation where all participants show little change. Reliable or clinically-significant change analyses can offer useful insights at an individual level. A reliable change index (RCI; [62]) determines whether individual change scores are statistically significant (greater than a difference that could have occurred due to random measurement error alone) and is commonly used with self-report scale data. It is computed by dividing the difference between the pretreatment and posttreatment scores (in this case, immediately pre-and post-CFI) by the standard error of the difference between the two scores. For this study, we calculated an RCI of 6.45 for positive affect (using Mpre = 20.42, Mpost = 22.50, SDpre = 7.76, α = 0.91) and an RCI of 8.41 for negative affect (using Mpre = 19.8, Mpost = 15.9, SDpre = 9.59,α = 0.90), which represents the minimum change score required to conclude statistical significance. Change scores for each participant at each session were calculated by subtracting pre-CFI affect from post-CFI affect, and were compared against the RCIs. For example, a client scoring 13 in positive affect pre-CFI and 20 post-CFI would have a change score of +7, representing a clinically-significant improvement (>6.45).

Reliable change is not suitable for analyzing HRV data since internal consistency cannot be calculated. Instead, we calculated how many individuals showed a clinically-significant HRV response for each session, which we defined as 5ms change in RMSSD during the experimental task (CFI or relaxation) compared to the control task. This value was drawn from literature on the differences in baseline levels of HRV between healthy controls and patients with depressive disorder [63, 64]. RMSSD provides an index in a common metric (milliseconds) and is well suited for use as a biomarker in a clinical setting [65]. The number of positive and negative clinically-significant responses were compared (i) between relaxation and compassion at session 1 in order to compare effects of both tasks, and (ii) over four sessions for both relaxation and for compassion, to evaluate whether repeated sessions improved response to each task. Facet wraps for both HRV and affect data were prepared in order to illustrate the variation in individual responses over time.

We tested the following hypotheses (H1) At session 1, group mean HRV will be lower during the control task than for both CFI and relaxation imagery; (H2) Examining individual HRV data, fewer individuals will show a clinically-significant negative response to compassion than to relaxation at each testing session (defined as 5ms lower than their HRV during a control task) and equally, more individuals will show a positive response to compassion than to relaxation; (3) Over time, the number of individuals showing a clinically-significant negative HRV response to CFI and relaxation will decrease, and finally (4) Regarding self-reported affect (which was only measured during CFI), group means will demonstrate a pre-post improvement during CFI sessions in positive affect. Due to previous conflicting findings we did not make a hypothesis regarding whether negative affect would significantly change.

## Results

### Hypothesis 1: At session 1, group mean HRV will be similar for CFI and relaxation imagery, but will be lower during the control task

A one-way ANOVA indicated no significant differences between RMSSD during the three tasks at session 1 (F(2,48) = 3.186, p = .05). Since the p-value was borderline (p = .0502), we chose to report post-hoc t-tests with effect sizes (see Table 1). Although the difference between self-compassion and relaxation RMSSD showed a medium effect-size (p = .038, d = .44), none of the comparisons were significant at the α = .01 level (chosen to correct for multiple testing).

**Table 1:** T-tests comparing RMSSD during three tasks.

*Pair*	*Variables*	*Mean*	*SD*	*t*	*df*	*p*	*95% CI*	*d*
***1***	***Self-compassion***	***61*.*80***	***40*.*29***	***2*.*200***	***24***	**.*038***	***1*.*01 to 31*.*72***	***0*.*44***
	***Relaxation***	***45*.*44***	***25*.*00***					
***2***	***Self-compassion***	***61*.*80***	***40*.*29***	***1*.*735***	***24***	**.*096***	***-2*.*53 to 29*.*19***	***0*.*34***
	***Control***	***48*.*48***	***32*.*65***					
***3***	***Relaxation***	***45*.*44***	***25*.*00***	**.*570***	***24***	**.*574***	***-7*.*95 to 14*.*02***	***0*.*11***
	***Control***	***48*.*48***	***32*.*65***					

### Hypothesis 2: Fewer individuals will show a clinically-significant negative HRV response to compassion than to relaxation at each testing session (defined as 5ms lower than their HRV during a control task) and equally, more individuals will show a positive response s to compassion than to relaxation; AND Hypothesis 3: Over time, the number of individuals showing a clinically-significant negative HRV response to CFI or relaxation will decrease

Although three participants dropped out after session 1, all of them showed a more positive physiological response to compassionate imagery compared to the control task. Thus, dropout does not seem to be associated with distress from the compassion task.

Table 2 presents the number of participants who showed a clinically significant change in HRV during CFI or relaxation (compared to control). Over time, for both CFI and relaxation imagery, the number of clinically significant positive responses decreased whilst the number of clinically significant negative responses increased, indicating that participants experienced less benefit to both exercises over time. CFI produced more clinically significant positive responses than did relaxation imagery, in all moments except session 3. At all sessions, CFI produced fewer or equal numbers of clinically significant negative responses than did relaxation imagery. Examining the four participants who had clinically significant negative HRV responses to CFI at session 1, two showed positive responses at their final session (P8 and P2), P5 showed a non-clinically-significant response, and P22 showed a negative response. This indicates that initial poor-responders can subsequently experience benefits from CFI, even though at a group level practice does not improve outcomes.

**Table 2:** Number and % of participants demonstrating a clinically-significant difference on RMSSD HRV (comparing compassion or relaxation task to control).

Session		1	2	3	4
Total N available		25	22	20^a^	8
CFI (compared to control condition)	N (%) of clinically significant positive responses	14 (56.0%)	9 (40.91%)	3 (15.0%)	3 (37.58%)
	N (%) of clinically significant negative responses	4 (16.0%)	5 (22.7%)	6 (30.0%)	2 (25.0%)
Relaxation (compared to control condition)	N (%) of clinically significant positive responses	11 (44.0%)	7 (31.8%)	5 (25.0%)	3 (37.5%)
	N (%) of clinically significant negative responses	7 (28.0%)	6 (27.3%)	6 (30.0%)	3 (37.5%)

Note: CFI = compassion-focused imagery.

^a^22 participants attended session 3 but their HRV data was corrupted (see Consort diagram) so could not be analysed.

Typically, the subset of individuals responding positively/negatively to CFI were similar to the subset of those responding positively/negatively to relaxation, suggesting that reasons for poor outcomes might be common to both tasks. For example, of the 11 who responded positively to relaxation at time 1, 10 had also responded positively to CFI. Of the 4 who responded negatively to CFI at time 1, all 4 also responded negatively to relaxation.

**Fig 3:**
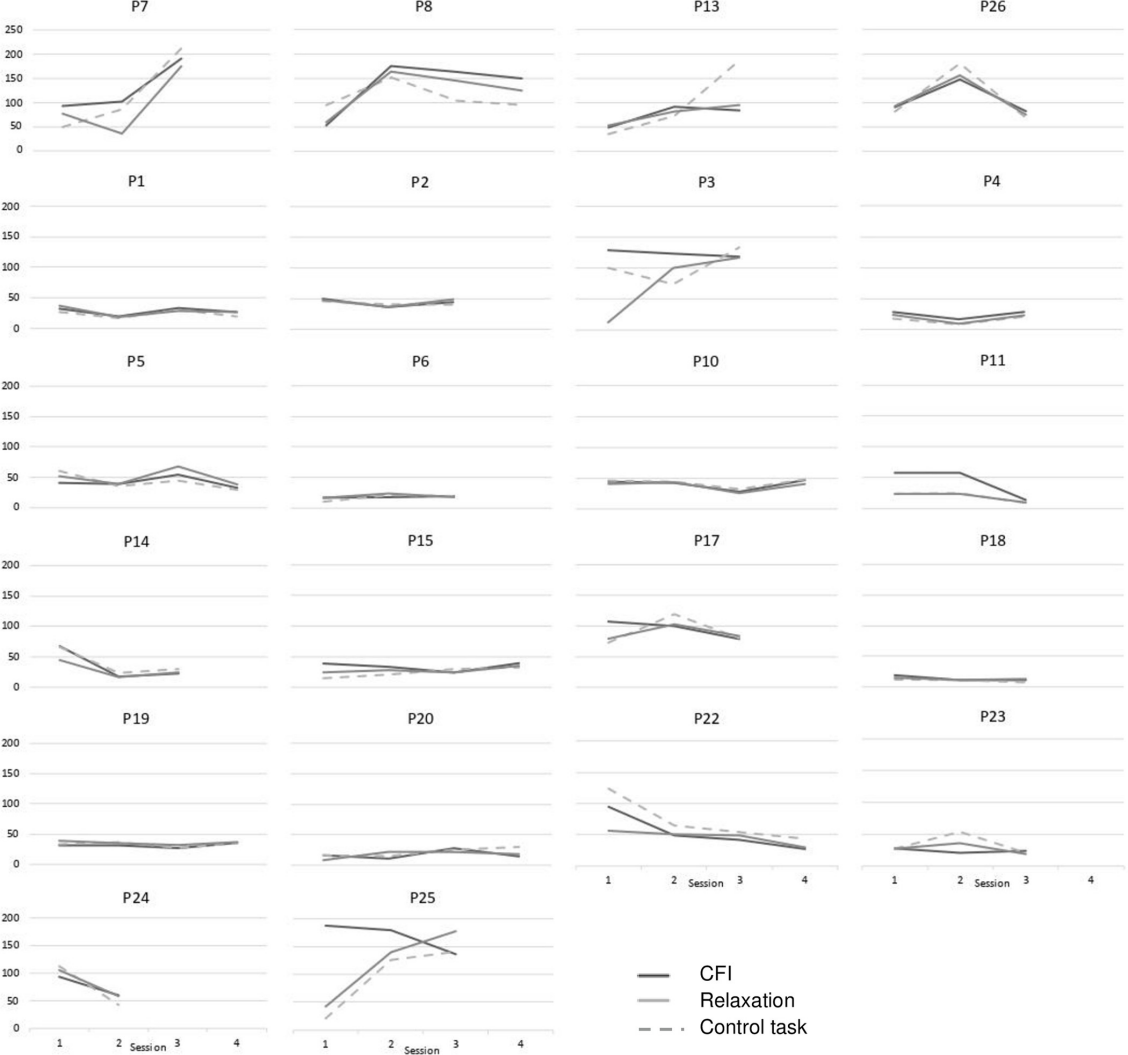
RMSSD HRV responses to CFI and control task over time, for participants who attended multiple sessions (N = 22). Note: X-axis represents sessions over time; Y-axis represents RMSSD HRV.

Fig 3 shows HRV responses to CFI, relaxation and the control task over time for the 22 participants who attended multiple sessions. Whilst some participants show considerable variance over time, much of this reflects task external factors such as sleep or food intake, since participants usually show this variance over time in all conditions (including the control task).

### Hypothesis 4: Group means will demonstrate a pre-post improvement during CFI sessions in positive affect. (No hypothesis was made regarding negative affect due to previous conflicting findings)

Self-report data indicated that across all participants, self-compassionate imagery had a small-to-medium-size effect on *positive* affect (*d* = 0.40), producing a close-to-significant increase (*t*(24) = -1.983, *p* = .059, 95% CI -5.38 to 0.11) from pre (M = 19.84, SD = 7.31) to post-imagery (M = 22.48, SD = 7.83). In contrast, CFI had a trivial effect on *negative* affect (*d* = 0.02), producing a non-significant increase (*t*(24) = 0.087, *p* = .931, 95% CI -4.95 to 4.55) from pre-(M = 24.04, SD = 12.58) to post-imagery (M = 24.24, SD = 12.88).

**Fig 4:**
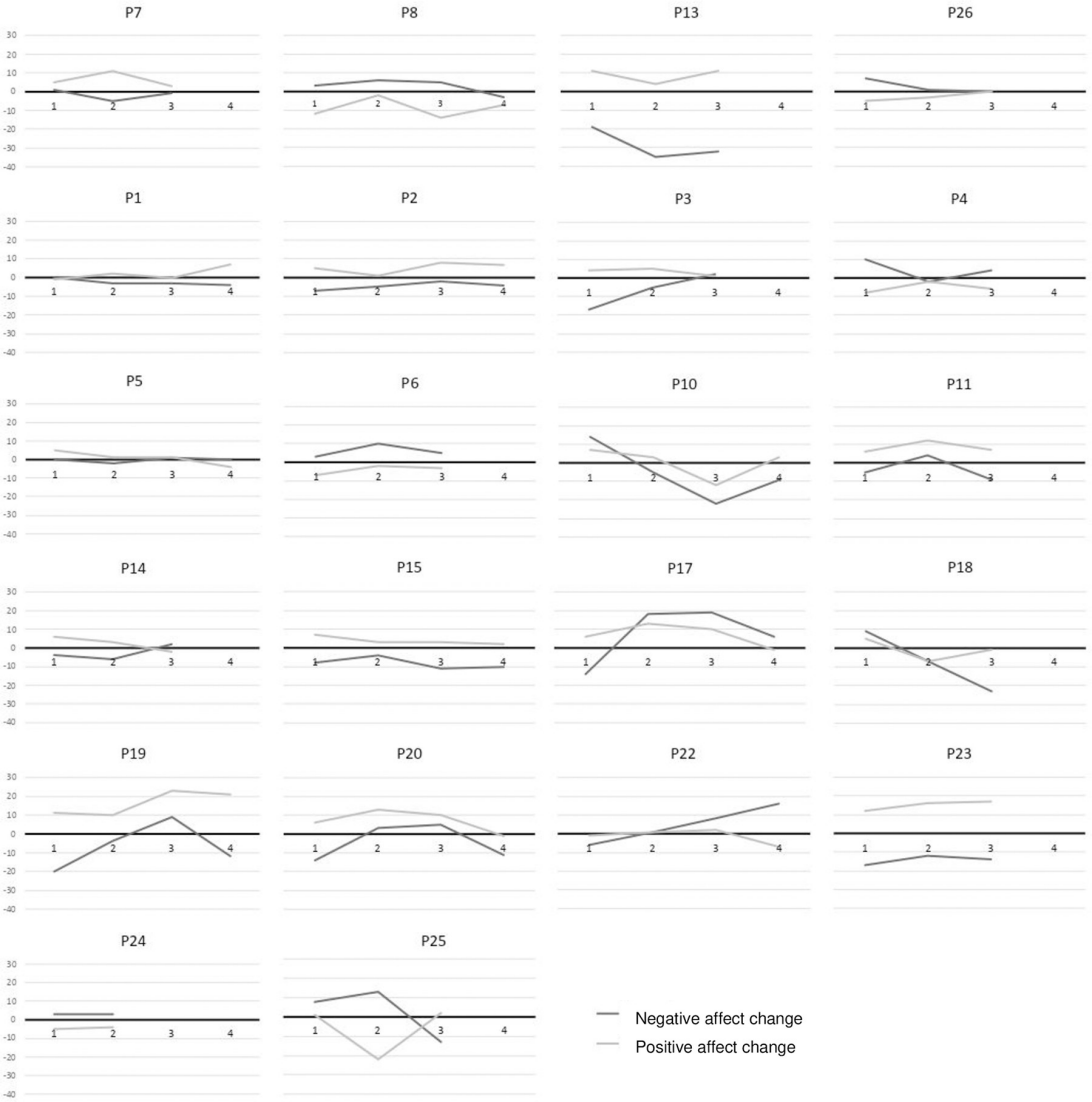
Self-reported affect change during CFI for participants who attended multiple sessions (N = 22). Note. X-axis represents sessions over time (one to four sessions); Y-axis represents self-reported affect change. Scores above 0 at any timepoint represent higher positive or negative affect after CFI than beforehand; thus, participants responding well to CFI will score above 0 for positive affect (grey line) and below 0 for negative affect (black line).

Fig 4 displays a facet wrap of self-report affect change scores during CFI, for all participants who attended multiple sessions. Findings indicated that the proportion of participants showing reliable change was relatively stable over time. Specifically, CFI was associated with a reliable increase in self-reported positive affect in 23.1% of participants at time 1, 21.7% of participants at time 2, and 26.1% of participants at time 3. It was associated with a reliable increase in negative affect in 11.5% of participants at time 1, 8.7% of participants at time 2, and 8.7% of participants at time 3.

## Discussion

The present study evaluated how a clinical population with high self-criticism and/or low self-compassion responded to self-compassionate imagery over time, compared to relaxation imagery and a control task. Hypotheses 1, 3 and 4 were not supported; but Hypothesis 2 was supported. Below, results are discussed in further detail.

Findings did not support Hypothesis 1 since at the initial session, no between-task differences were seen (although a trend in the expected direction was seen for self-compassion, which produced higher HRV than both relaxation and control tasks). The non-significant findings may reflect the small sample size, high variance of HRV between participants, and the fact that some individuals in session 1 responded positively to the two therapeutic tasks and others neutrally or negatively (see Table 2), thus producing non-significant differences at a group-level analysis. This corroborates previous studies in that whilst relaxation and compassion may stimulate the parasympathetic system in some individuals, it may produce a threat response in others, at least in clinical samples.

Hypothesis 2 was supported, since at each session a greater number of individuals showed positive responses to both compassion and to relaxation, and fewer number of individuals showed a negative response to compassion compared to relaxation. Specifically, at the first assessment, clinically-significant increases in HRV was found in 56% of participants, which was more than the proportion that showed a clinically-significant positive response to relaxation imagery (44%). Furthermore, fewer participants showed a clinically-significant negative response to self-compassionate imagery (16%) than to relaxation (28%).

Together, these results indicate that self-compassion imagery is at least as acceptable as commonly-used relaxation exercises—even for individuals with characteristics that make them most at risk for showing threat-responses to compassion [55]. Thus, whilst clinicians should be alert to possible threat-responses to compassion-focused imagery, our preliminary results suggest that a short self-compassion imagery is less threat-provoking than relaxation, another widely used therapeutic task. Other studies have also noted that relaxation can occasionally be threatening and generate an aversive experience of loss of control [66].

Given that there were no significant differences at the group level, we cannot hypothesize whether the mechanisms that triggered a positive or negative response in clients are the same or different across the two therapeutic tasks of compassion and relaxation. CFI has been hypothesized to specifically activate care-based motivation of compassion, rooted in evolutionarily developed brain systems that humans share with other mammals and linked to the myelinated vagus nerve [67, 68], which in turn inhibits sympathetically driven threat-defensive behaviours (e.g., fight/flight) and hypothalamic-pituitary-adrenal (HPA) axis activity [14, 15]. In comparison, relaxation does not necessarily comprise elements of affiliation and caring. Nonetheless, further physiological and neuropsychological research with larger samples will be required to determine the exact impact of each task on the brain and body.

### Responses over time

In contrast to Hypothesis 3, for both compassion and relaxation exercises, HRV data indicated a decreasing number of clinically-significant positive responses over the 3–4 sessions and an increasing number of reliably negative responses. This trend was more pronounced for CFI than relaxation. One possible explanation for this result is that it reflects boredom due to habituation, which may have been lower in the relaxation condition since it used forest and beach scenes alternately, whilst the CFI task was left unchanged. Clinicians might consider helping clients try different variations of a therapy task to avoid habituation during repeated homework sessions; although admittedly, boredom or frustration may have been inflated in the current context due to doing the task at pre-scheduled hours, rather than when a client might choose to practice. Repeated exposures seem not to be effective in reducing threat responses that might potentially arise during self-compassion imagery. This finding is in line with compassion-focused psychotherapy approaches, such as Compassion Focused Therapy, which highlights the importance of helping the client understand the nature and the origin of the resistances to specific flows of compassion and work compassionately with these inhibitors [30].

### Self-reported affect outcomes

Regarding hypothesis 4, self-compassionate imagery was not associated with a significant difference in negative or positive affect in session 1. Nonetheless, the effect size for positive affect was small-to-medium-sized and showed a trend in the expected direction. Larger samples might be useful to test this further.

Self-report affect data largely indicated little change over sequential sessions, corroborating the physiological data discussed previously. As Fig 4 illustrates, most participants remained relatively stable over time, including those who responded well to CFI, as defined by self-reported increases in positive affect and decreases in negative affect during the exercise (P2, P11, P13, P15, P17, P19, and P23); as well as those who responded poorly (P4, P6, P24). A few showed worsening response over time (P3, P14, P20, P22), with more pronounced changes in negative affect than in positive affect, which may reflect boredom due to habituation as previously discussed. A minority showed improvement over time (P10, P21, P25), again with more pronounced changes in negative affect, which might reflect a decreased threat response.

### Limitations

The sample was small, relatively young and predominantly female, reflecting the study´s university location and cultural gender norms of help-seeking. Findings are therefore preliminary and replication with a larger and more representative sample is warranted. Whilst positive affect items were drawn from a validated measure, the negative affect measure was not, since validated measures of state affect either measure overly-generic negative affect [69], or measure fear with words that may be too intense to pick up changes in this study, e.g. “scared”, “frightened”, “afraid” [70]. As noted previously, self-report affect measures were not applied during relaxation to avoid participant fatigue, thus cannot be used to compare tasks.

The response to the control task (reading a city culture magazine) varied considerably between sessions (see Fig 3**),** which might reflect varying ability to concentrate on the task or be motivated by it, as well as task-external factors (food intake, sleep). It is unclear whether a different control task would provide a more constant baseline. This study specifically selected a compassion task involving only *self-to-self* relating. Results cannot be generalized to tasks involving (imagining) receiving compassion from others or giving to others, as these tasks may trigger other fears or inhibitors. All participants received compassion following relaxation to prevent compassion contaminating the relaxation task, since compassion includes elements of relaxation but not vice versa. Therefore, when comparing relaxation and compassion directly, it should be acknowledged that the compassion task may reflect this.

The compassion task in our study focused on willing oneself to be calm and well, in line with loving-kindness meditations [71] and alluded to general suffering (reminding participants that “life can be difficult”, “we all make mistakes”, and suggesting they “tolerate your difficulties and emotions”). However, it did not ask participants to bring to mind a specific moment of suffering, to avoid introducing significant negative affect not triggered by the relaxation exercise. Some key compassion thinkers (e.g. Paul Gilbert, Thupten Jinpa) argue that a key element of compassion is awareness of suffering [71], thus, our findings may differ from exercises that include this element. Interestingly, one study found self-compassion to be more effective under higher distress because participants felt more deserving of it [72], so including awareness of suffering might actually enhance compassion effectiveness.

## Conclusions

Threat-based responses to compassionate exercises are sometimes viewed as a reason to not assign them to clients. The present study offers preliminary evidence that self-compassion is no more threatening than relaxation imagery, a task which is widely applied without therapeutic groundwork, even for clients most likely to respond poorly to compassion. It may be that non-specific elements such as attending to breath or conjuring mental imagery may sometimes be at the root of these negative responses. Repeated practice of the same exercise often produces unchanged or worsening responses, rather than improvement. Habituation to exercises may be lessened if clients can personalize exercises or alternate between different ones, as in real therapy contexts. However, these findings also emphasize the need to respond in novel ways when clients struggle with compassion, since prescribing continued practice is unlikely to help. Current available techniques include guided discovery around inhibitors, exploration of past traumatic experiences, and practice of other supporting skills such as mindfulness (e.g. Gilbert, 2009). Further research will be able to illuminate the most effective ways to overcome these inhibitors, depending on client needs.

## Supporting information

S1 Checklist (DOCX)

S1 Text Script for self-compassionate imagery task. (DOCX)

S1 File (DOCX)

S2 File (DOCX)

## References

[1] Werner AM, Tibubos AN, Rohrmann S, Reiss N. The clinical trait self-criticism and its relation to psychopathology: A systematic review–Update. J Affect Disord [Internet]. 2019 Mar;246:530–47. Available from: https://linkinghub.elsevier.com/retrieve/pii/S0165032718312254 doi: 10.1016/j.jad.2018.12.069 30599378

[2] Longe O, Maratos FA, Gilbert P, Evans G, Volker F, Rockliff H, et al. Having a word with yourself: Neural correlates of self-criticism and self-reassurance. Neuroimage [Internet]. 2010 Jan;49(2):1849–56. Available from: https://linkinghub.elsevier.com/retrieve/pii/S1053811909009987 doi: 10.1016/j.neuroimage.2009.09.019 19770047

[3] Gilbert P, Procter S. Compassionate mind training for people with high shame and self-criticism: overview and pilot study of a group therapy approach. Clin Psychol Psychother [Internet]. 2006 Nov;13(6):353–79. Available from: http://doi.wiley.com/ doi: 10.1002/cpp.507

[4] Krieger T, Reber F, von Glutz B, Urech A, Moser CT, Schulz A, et al. An Internet-Based Compassion-Focused Intervention for Increased Self-Criticism: A Randomized Controlled Trial. Behav Ther [Internet]. 2019 Mar;50(2):430–45. Available from: https://linkinghub.elsevier.com/retrieve/pii/S0005789418301060 doi: 10.1016/j.beth.2018.08.003 30824257

[5] Kirby JN, Tellegen CL, Steindl SR. A Meta-Analysis of Compassion-Based Interventions: Current State of Knowledge and Future Directions. Behav Ther [Internet]. 2017 Nov;48(6):778–92. Available from: https://linkinghub.elsevier.com/retrieve/pii/S0005789417300667 doi: 10.1016/j.beth.2017.06.003 29029675

[6] Gilbert P. The origins and nature of compassion focused therapy. Br J Clin Psychol. 2014;53(1):6–41. doi: 10.1111/bjc.12043 24588760

[7] Andrews B. Shame and childhood abuse. In: Gilbert P, Andrews B, editors. Shame: Interpersonal behavior, psychopathology, and culture. Oxford University Press; 1998. p. 176–90.

[8] Schore AN. Early shame experiences and infant brain development. In: Gilbert P, Andrews B, editors. Shame: Interpersonal behavior, psychopathology, and culture. New York: Oxford University Press; 1998. p. 57–77.

[9] Bowlby J. Attachment: Attachment and loss (Vol. I). London: Hogarth Press; 1969.

[10] Kohut H. The restoration of the self. New York: International Universities Press; 1977.

[11] Naismith I, Zarate Guerrero S, Feigenbaum J. Abuse, invalidation, and lack of early warmth show distinct relationships with self‐criticism, self‐compassion, and fear of self‐compassion in personality disorder. Clin Psychol Psychother [Internet]. 2019 May 27;26(3):350–61. Available from: https://onlinelibrary.wiley.com/doi/abs/10.1002/cpp.2357 30715768 10.1002/cpp.2357

[12] Boersma K, Håkanson A, Salomonsson E, Johansson I. Compassion Focused Therapy to Counteract Shame, Self-Criticism and Isolation. A Replicated Single Case Experimental Study for Individuals With Social Anxiety. J Contemp Psychother [Internet]. 2015 Jun 6;45(2):89–98. Available from: http://link.springer.com/10.1007/s10879-014-9286-8

[13] Gilbert P, McEwan K, Mitra R, Franks L, Richter A, Rockliff H. Feeling safe and content: A specific affect regulation system? Relationship to depression, anxiety, stress, and self-criticism. J Posit Psychol [Internet]. 2008 Jul;3(3):182–91. Available from: http://www.tandfonline.com/doi/abs/10.1080/17439760801999461

[14] Petrocchi N, Cheli S. The social brain and heart rate variability: Implications for psychotherapy. Psychol Psychother Theory, Res Pract [Internet]. 2019 Jun 20;92(2):208–23. Available from: https://onlinelibrary.wiley.com/doi/abs/10.1111/papt.12224 30891894 10.1111/papt.12224

[15] Porges SW. The polyvagal perspective. Biol Psychol [Internet]. 2007 Feb;74(2):116–43. Available from: https://linkinghub.elsevier.com/retrieve/pii/S0301051106001761 doi: 10.1016/j.biopsycho.2006.06.009 17049418 PMC1868418

[16] Gilbert P. Compassion focused therapy: Distinctive features. London: Routledge; 2010.

[17] Holmes EA, Mathews A. Mental Imagery and Emotion: A Special Relationship? Emotion [Internet]. 2005;5(4):489–97. Available from: http://doi.apa.org/getdoi.cfm?doi=10.1037/1528-3542.5.4.489 16366752 10.1037/1528-3542.5.4.489

[18] Lincoln TM, Hohenhaus F, Hartmann M. Can Paranoid Thoughts be Reduced by Targeting Negative Emotions and Self-Esteem? An Experimental Investigation of a Brief Compassion-Focused Intervention. Cognit Ther Res [Internet]. 2013 Apr 14;37(2):390–402. Available from: http://link.springer.com/10.1007/s10608-012-9470-7

[19] Petrocchi N, Ottaviani C, Couyoumdjian A. Compassion at the mirror: Exposure to a mirror increases the efficacy of a self-compassion manipulation in enhancing soothing positive affect and heart rate variability. J Posit Psychol [Internet]. 2017 Nov 2;12(6):525–36. Available from: https://www.tandfonline.com/doi/full/10.1080/17439760.2016.1209544

[20] Rockliff H, Gilbert P, McEwan K, Lightman S, Glover D. A pilot exploration of heart rate variability and salivary cortisol responses to compassion-focused imagery. 2008; Clinical Neuropsychiatry: Journal of Treatment Evaluation, 5(3), 132–139.

[21] Gilbert P, Irons C. A pilot exploration of the use of compassionate images in a group of self‐critical people. Memory [Internet]. 2004 Jul;12(4):507–16. Available from: http://www.tandfonline.com/doi/abs/10.1080/09658210444000115 15487546 10.1080/09658210444000115

[22] McEwan K, Gilbert P. A pilot feasibility study exploring the practising of compassionate imagery exercises in a nonclinical population. Psychol Psychother Theory, Res Pract [Internet]. 2016 Jun;89(2):239–43. Available from: http://doi.wiley.com/ doi: 10.1111/papt.12078 26454144

[23] Naismith I, Mwale A, Feigenbaum J. Inhibitors and facilitators of compassion-focused imagery in personality disorder. Clin Psychol Psychother [Internet]. 2018 Mar;25(2):283–91. Available from: http://doi.wiley.com/10.1002/cpp.2161 29251381 10.1002/cpp.2161

[24] Klimecki OM, Leiberg S, Lamm C, Singer T. Functional Neural Plasticity and Associated Changes in Positive Affect After Compassion Training. Cereb Cortex [Internet]. 2013 Jul;23(7):1552–61. Available from: https://academic.oup.com/cercor/article-lookup/doi/10.1093/cercor/bhs142 22661409 10.1093/cercor/bhs142

[25] Duarte J, McEwan K, Barnes C, Gilbert P, Maratos FA. Do therapeutic imagery practices affect physiological and emotional indicators of threat in high self-critics? Psychol Psychother Theory, Res Pract [Internet]. 2015 Sep;88(3):270–84. Available from: http://doi.wiley.com/10.1111/papt.12043 25347984 10.1111/papt.12043

[26] Lawrence VA, Lee D. An Exploration of People’s Experiences of Compassion-focused Therapy for Trauma, Using Interpretative Phenomenological Analysis. Clin Psychol Psychother [Internet]. 2013 Jul;n/a-n/a. Available from: http://doi.wiley.com/10.1002/cpp.1854 23893917 10.1002/cpp.1854

[27] Lucre KM, Corten N. An exploration of group compassion-focused therapy for personality disorder. Psychol Psychother Theory, Res Pract [Internet]. 2013 Dec;86(4):387–400. Available from: http://doi.wiley.com/10.1111/j.2044-8341.2012.02068.x 24217864 10.1111/j.2044-8341.2012.02068.x

[28] McLean L, Bambling M, Steindl SR. Perspectives on Self-Compassion From Adult Female Survivors of Sexual Abuse and the Counselors Who Work With Them. J Interpers Violence [Internet]. 2018 Aug 22;088626051879397. Available from: http://journals.sagepub.com/doi/10.1177/0886260518793975 10.1177/0886260518793975 30132732

[29] Naismith I, Kerr S, Mwale A, Feigenbaum J. A thematic analysis of compassion‐focused imagery for people with personality disorder: Inhibitors, facilitators and clinical recommendations. Clin Psychol [Internet]. 2019 Nov 4;23(3):213–24. Available from: https://onlinelibrary.wiley.com/doi/abs/10.1111/cp.12180

[30] Gilbert P, McEwan K, Matos M, Rivis A. Fears of compassion: Development of three self-report measures. Psychol Psychother Theory, Res Pract [Internet]. 2011 Sep;84(3):239–55. Available from: http://doi.wiley.com/10.1348/147608310X526511 22903867 10.1348/147608310X526511

[31] Gilbert P, Catarino F, Duarte C, Matos M, Kolts R, Stubbs J, et al. The development of compassionate engagement and action scales for self and others. J Compassionate Heal Care [Internet]. 2017 Dec 27;4(1):4. Available from: http://jcompassionatehc.biomedcentral.com/articles/10.1186/s40639-017-0033-3

[32] De Wet A. J., Lane B. R., & Mulgrew K. E. (2020). A Randomised Controlled Trial Examining the Effects of Self-Compassion Meditations on Women’s Body Image. Body Image, 35, 22–29. doi: 10.1016/j.bodyim.2020.07.009 32846388

[33] Halamová, Júlia; Kanovský, Martin; Koróniová, Jana (2019). Heart Rate Variability Differences among Participants with Different Levels of Self-Criticism during Exposure to a Guided Imagery. Adaptive Human Behavior and Physiology, 5(4), 371–381. doi: 10.1007/s40750-019-00122-3

[34] Germer CK, Neff KD. Self‐compassion in clinical practice. J Clin Psychol. 2013;69(8):856–67. doi: 10.1002/jclp.22021 23775511

[35] Robinson KJ, Mayer S, Allen AB, Terry M, Chilton A, Leary MR. Resisting self-compassion: Why are some people opposed to being kind to themselves? Self Identity [Internet]. 2016 Sep 2;15(5):505–24. Available from: http://www.tandfonline.com/doi/full/10.1080/15298868.2016.1160952

[36] Nicolò G, Semerari A, Lysaker PH, Dimaggio G, Conti L, D’Angerio S, et al. Alexithymia in personality disorders: Correlations with symptoms and interpersonal functioning. Psychiatry Res [Internet]. 2011 Nov;190(1):37–42. Available from: https://linkinghub.elsevier.com/retrieve/pii/S0165178110004786 doi: 10.1016/j.psychres.2010.07.046 20800288

[37] Thayer JF, Lane RD. Claude Bernard and the heart–brain connection: Further elaboration of a model of neurovisceral integration. Neurosci Biobehav Rev [Internet]. 2009 Feb;33(2):81–8. Available from: https://linkinghub.elsevier.com/retrieve/pii/S0149763408001255 doi: 10.1016/j.neubiorev.2008.08.004 18771686

[38] Thayer JF, Lane RD. A model of neurovisceral integration in emotion regulation and dysregulation. J Affect Disord [Internet]. 2000 Dec;61(3):201–16. Available from: https://linkinghub.elsevier.com/retrieve/pii/S0165032700003384 doi: 10.1016/s0165-0327(00)00338-4 11163422

[39] Kirby JN, Doty JR, Petrocchi N, Gilbert P. The Current and Future Role of Heart Rate Variability for Assessing and Training Compassion. Front Public Heal [Internet]. 2017 Mar 8;5. Available from: http://journal.frontiersin.org/article/10.3389/fpubh.2017.00040/full 28337432 10.3389/fpubh.2017.00040 PMC5340770

[40] Kok BE, Coffey KA, Cohn MA, Catalino LI, Vacharkulksemsuk T, Algoe SB, et al. How positive emotions build physical health: perceived positive social connections account for the upward spiral between positive emotions and vagal tone. *Psychol Sci* (2013) 24:1123–32. doi: 10.1177/0956797612470827 23649562

[41] Krygier JR, Heathers JA, Shahrestani S, Abbott M, Gross JJ, Kemp AH. Mindfulness meditation, well-being, and heart rate variability: a preliminary investigation into the impact of intensive Vipassana meditation. *Int J Psychophysiol* (2013) 89:305–13. doi: 10.1016/j.ijpsycho.2013.06.017 23797150

[42] Korpal P, Jankowiak K. Physiological and self-report measures in emotion studies: Methodological considerations. Polish Psychological Bulletin. 2018;49(4):475–481.

[43] Norman SB, Hami Cissell S, Means-Christensen AJ, Stein MB. Development and validation of an Overall Anxiety Severity And Impairment Scale (OASIS). Depress Anxiety [Internet]. 2006;23(4):245–9. Available from: http://doi.wiley.com/10.1002/da.20182 16688739 10.1002/da.20182

[44] Silva Carvajal L, Unda McFarlane J. Analisis de confiabilidad de la adaptación del overall anxiety and impairment scale (OASIS) y el overall depression and impairment scale (ODSIS) al el contexto colombiano [Internet]. Repositorio.uniandes.edu.co. 2015. Available from: https://repositorio.uniandes.edu.co/bitstream/handle/1992/17457/u713637.pdf?sequence=1

[45] Bentley KH, Gallagher MW, Carl JR, Barlow DH. Development and validation of the Overall Depression Severity and Impairment Scale. Psychol Assess [Internet]. 2014 Sep;26(3):815–30. Available from: http://doi.apa.org/getdoi.cfm?doi=10.1037/a0036216 24708078 10.1037/a0036216

[46] Hirschfeld R. M., Williams J. B., Spitzer R. L., Calabrese J. R., Flynn L., Keck P. E. Jr, et al. (2000). Development and validation of a screening instrument for bipolar spectrum disorder: the Mood Disorder Questionnaire. *The American journal of psychiatry*, 157(11), 1873–1875. 10.1176/appi.ajp.157.11.1873 11058490

[47] Moran P., Leese M., Lee T., Walters P., Thornicroft G., & Mann A. (2003). Standardised Assessment of Personality–Abbreviated Scale (SAPAS): preliminary validation of a brief screen for personality disorder. British Journal of Psychiatry 183: 228–232. doi: 10.1192/bjp.183.3.228 12948996

[48] Gossop M, Darke S, Griffiths P, Hando J., Powis B., Hall W, et al. (1995). The Severity of Dependence Scale (SDS): psychometric properties of the SDS in English and Australian samples of heroin, cocaine and amphetamine users. Addiction, 90(5):607–14. doi: 10.1046/j.1360-0443.1995.9056072.x 7795497

[49] BIOPAC Systems, Inc., Goleta, CA, United States.

[50] Kubios version 3.3, 2019, Biosignal Analysis and Medical Imaging Group, University of Kuopio, Finland, MATLAB.

[51] Niskanen J-P, Tarvainen MP, Ranta-aho PO, Karjalainen PA. Software for advanced HRV analysis. Comput Methods Programs Biomed [Internet]. 2004 Oct;76(1):73–81. Available from: https://linkinghub.elsevier.com/retrieve/pii/S0169260704000719 10.1016/j.cmpb.2004.03.004 15313543

[52] Malik M, Bigger JT, Camm AJ, Kleiger RE, Malliani A, Moss AJ, et al. Heart rate variability: Standards of measurement, physiological interpretation, and clinical use. Eur Heart J [Internet]. 1996 Mar 1;17(3):354–81. Available from: https://academic.oup.com/eurheartj/article-lookup/doi/10.1093/oxfordjournals.eurheartj.a014868 8737210

[53] Gilbert P, Clarke M, Hempel S, Miles JNV, Irons C. Criticizing and reassuring oneself: An exploration of forms, styles and reasons in female students. Br J Clin Psychol [Internet]. 2004 Mar;43(1):31–50. Available from: http://doi.crossref.org/10.1348/014466504772812959 15005905 10.1348/014466504772812959

[54] López Cavada C, Hornillos Cárdenas T, López-Romero HY. Self-criticism: measure and Treatment. Int Soc Emot Focus Ther (ISEFT), Toronto. 2017.

[55] Naismith I, Duran Ferro C, Ingram G, Jimenez Leal W. Compassion-focused imagery reduces shame and is moderated by shame, self-reassurance and multisensory imagery vividness. Res Psychother Psychopathol Process Outcome [Internet]. 2019 Jan 17;22(1). Available from: https://www.researchinpsychotherapy.org/index.php/rpsy/article/view/329 doi: 10.4081/ripppo.2019.329 32913776 PMC7451339

[56] Sanchez-Moreno J, Villagran J, Gutierrez J, Camacho M, Ocio S, Palao D et al. Adaptation and validation of the Spanish version of the Mood Disorder Questionnaire for the detection of bipolar disorder. Bipolar Disorders. 2008;10(3):400–412. doi: 10.1111/j.1399-5618.2007.00571.x 18402628

[57] Germans S, Van Heck GL, Moran P, Hodiamont PPG: The self-report Standardized Assessment of Personality–Abbreviated Scale: preliminary results of a brief screening test for personality disorders. Personal Ment Health 2008;2:70–76.

[58] Lawrinson P., Copeland J., Gerber S., & Gilmour S. (2007). Determining a cut-off on the Severity of Dependence Scale (SDS) for alcohol dependence. *Addictive Behaviors*, 32(7), 1474–1479. doi: 10.1016/j.addbeh.2006.09.005 17081703

[59] Vélez-Moreno A, González-Saiz F, Rojas A, Torrico-Linares E, Fernández-Calderón F, Ramírez-López J et al. Reliability and Validity of the Spanish Version of the Substance Dependence Severity Scale. European Addiction Research. 2014;21(1):39–46. doi: 10.1159/000365282 25376716

[60] Linehan M., Comtois K., & Ward-Ciesielski E. (2012). Assessing and Managing Risk with Suicidal Individuals. *Cognitive And Behavioral Practice*, 19(2), 218–232. doi: 10.1016/j.cbpra.2010.11.008

[61] IBM Corp. Released 2016. IBM SPSS Statistics for Windows, Version 24.0. Armonk, NY: IBM Corp.

[62] Jacobson N, Truax PN. Clinical significance: a statistical approach to defining meaningful change in psychotherapy research. Journal of Consulting and Clinical Psychology [Internet]. 1991; 59(1), 12–19. Available from: doi: 10.1037//0022-006x.59.1.12 2002127

[63] Hu MX, Lamers F, de Geus EJC, Penninx BWJH. Differential Autonomic Nervous System Reactivity in Depression and Anxiety During Stress Depending on Type of Stressor. Psychosom Med [Internet]. 2016 Jun;78(5):562–72. Available from: http://journals.lww.com/00006842-201606000-00007 doi: 10.1097/PSY.0000000000000313 26910796

[64] Moon E, Lee S-H, Kim D-H, Hwang B. Comparative Study of Heart Rate Variability in Patients with Schizophrenia, Bipolar Disorder, Post-traumatic Stress Disorder, or Major Depressive Disorder. Clin Psychopharmacol Neurosci [Internet]. 2013 Dec 28;11(3):137–43. Available from: http://www.cpn.or.kr/journal/view.html?doi=10.9758/cpn.2013.11.3.137 24465250 10.9758/cpn.2013.11.3.137 PMC3897762

[65] Jarczok MN, Koenig J, Wittling A, Fischer JE, Thayer JF. First Evaluation of an Index of Low Vagally-Mediated Heart Rate Variability as a Marker of Health Risks in Human Adults: Proof of Concept. J Clin Med [Internet]. 2019 Nov 11;8(11):1940. Available from: https://www.mdpi.com/2077-0383/8/11/1940 doi: 10.3390/jcm8111940 31717972 PMC6912519

[66] Heide FJ, Borkovec TD. Relaxation-induced anxiety: Mechanisms and theoretical implications. Behav Res Ther [Internet]. 1984;22(1):1–12. Available from: https://linkinghub.elsevier.com/retrieve/pii/0005796784900275 doi: 10.1016/0005-7967(84)90027-5 6365071

[67] Depue RA, Morrone-Strupinsky J V. A neurobehavioral model of affiliative bonding: Implications for conceptualizing a human trait of affiliation. Behav Brain Sci [Internet]. 2005 Jun 7;28(03). Available from: http://www.journals.cambridge.org/abstract_S0140525X05000063 doi: 10.1017/S0140525X05000063 16209725

[68] Baumeister RF, Bushman B. Social psychology and human nature. Belmonth, CA: Thomson Wadsworth; 2010.

[69] Watson D, Clark L, Tellegen A. Development and validation of brief measures of positive and negative affect: The PANAS scales. Journal of Personality and Social Psychology. 1988;54(6):1063–1070. doi: 10.1037//0022-3514.54.6.1063 3397865

[70] Watson D, Clark LA. The PANAS-X: Manual for the positive and negative affect schedule-Expanded Form. Iowa City: University of Iowa; 1994.

[71] Kirby James N. (2017). Compassion interventions: The programmes, the evidence, and implications for research and practice. Psychology and Psychotherapy: Theory, Research and Practice, 90(3), 432–455. doi: 10.1111/papt.12104 27664071

[72] Diedrich Alice; Grant Michaela; Hofmann Stefan G.; Hiller Wolfgang; Berking Matthias(2014). Self-compassion as an emotion regulation strategy in major depressive disorder. Behaviour Research and Therapy, 58(), 43–51. doi: 10.1016/j.brat.2014.05.006 24929927

