# Supplementary material for: Physiological response to self-compassion versus relaxation in a clinical population
Source: PLoS One. 2023 Feb 7;18(2):e0272198. doi: 10.1371/journal.pone.0272198 (PMC9904495; doi:10.1371/journal.pone.0272198)
Supplement: S1 Text — (DOCX) [file pone.0272198.s002.docx]

**Supplementary Material 1**

*Script for self-compassionate imagery task (english translation)*

Acting towards ourselves in an affectionate, kind and compassionate manner is a way of managing difficult emotions. In this exercise, we will try to develop several characteristics of compassion. It doesn't matter if you think you have these characteristics or not; the important thing is to imagine that you do. Please sit in a comfortable position. Let your eyes close, fully or partially. Take a few deep breaths to settle into your body and into the present moment. Put your hand over your chest, heart, or wherever it feels comforting. Gently bring your awareness onto yourself. Now become aware of your breath and try to breathe slowly. Feel yourself breathing in and out. When your attention wanders, gently focus on your breath again.

First, imagine that you are a very, very wise person. You know that life can be difficult and that this is not your fault. We all make mistakes. Remember that when we make a mistake, the most helpful thing is to motivate ourselves to change in a kind way, and not through blaming or criticizing ourselves about actions that are in the past.

Second, imagine that you have the strength to tolerate your difficulties and emotions, and the confidence to accept them without judging yourself.

Third, imagine you have a very warm and affectionate way of treating yourself. Imagine talking to yourself with warmth and kindness. Try to develop a kind facial expression while doing this, perhaps with a gentle smile.

Finally, commit to be there for yourself, to support yourself. Even in the most difficult situations, commit to not judging or blaming yourself for your mistakes. Instead, you help and give yourself what you need.

Keep breathing slowly. Offer yourself the following words of kindness and compassion and repeat them gently, feeling their importance:

- May you be happy (you might wish to use your name)
- May you be at peace
- May you be well
- May you be tranquil

Finally, take a few deep breaths and try to enjoy any feeling of calmness or tranquility that may arise. When you are ready, gently open your eyes.

*Guion para tarea de visualización autocompasiva (original en español)*

Actuando hacia nosotros de una manera afectuosa, amable y compasiva es una manera de manejar las emociones difíciles. Intentaremos desarrollar varias características compasivas en este ejercicio. No importa si piensas que tienes estas características o no; lo importante es imaginar que las tienes. Por favor, siéntate en una posición cómoda. Deja que tus ojos se cierren, total o parcialmente. Respira profundamente para acomodarte a tu cuerpo y al momento presente. Pon tu mano en el pecho, sobre tu corazón, o donde sea cómodo. Con amabilidad enfoca tu atención en ti mismo. Ahora hazte consciente de tu respiración e intenta respirar lentamente. Siente como tu cuerpo inhala y exhala, y si tu atención se dispersa, vuelve a enfocarte en tu respiración otra vez.

Primero, imagínate que eres una persona muy muy sabia. Sabes que la vida puede ser difícil sin que esto sea tu culpa, todos cometemos errores. Si cometes un error, te motivas a cambiar de manera amable, y no a través de culparte o criticarte sobre acciones que ya hiciste.

Segundo, imagínate que tienes la fuerza para tolerar tus dificultades y emociones, y la confianza de aceptarlos sin juzgarte.

Tercero, imagínate que tienes una manera muy cálida y afectuosa de tratarte a ti mismo. Imagínate hablándote con cálidad y amabilidad. Intenta tener una expresión facial amable mientras lo haces, tal vez con una sonrisa amable.

Finalmente, te comprometes a estar ahí para ti, a ser tu apoyo. Aún en las situaciones más dificiles te comprometes a no juzgarte y no a culparte por tus errores. Imagínate siendo tu propio apoyo, y estar ahí para ti. Aún en las situaciones más difíciles, te comprometes a no juzgarte y no ha culparte por tus errores, en cambio e ayudas y te das lo que necesitas.

Sigue respirando lentamente. Repite en tu mente las siguientes palabras de compasión, de manera amable y cálida, sintiendo su importancia.

o Quiero que seas feliz. (Usar tu nombre podría facilitar el ejercicio)

o Quiero que estés en paz.

o Quiero que tengas salud y bienestar.

o Quiero que vivas tranquilamente.

Por último, respira varias veces profundamente y simplemente trata de disfrutar cualquier sentimiento de tranquilidad que pueda surgir. Lentamente, abre tus ojos.
