# Supplementary material for: Physiological response to self-compassion versus relaxation in a clinical population
Source: PLoS One. 2023 Feb 7;18(2):e0272198. doi: 10.1371/journal.pone.0272198 (PMC9904495; doi:10.1371/journal.pone.0272198)
Supplement: S1 File — (DOCX) [file pone.0272198.s003.docx]

**FORMATO DE PRESENTACIÓN DE PROYECTOS AL COMITÉ DE ÉTICA**

**FACULTAD DE CIENCIAS SOCIALES**

| **TÍTULO PROYECTO: Exploración de los inhibidores de la compasión en una población clínica** |
| --- |
| **DEPARTAMENTO: Psicología** |
| **INVESTIGADOR(A): Iona Naismith** |
| **FECHA INICIO INVESTIGACIÓN: 15 de marzo de 2018** |

| **RESUMEN EJECUTIVO DEL PROYECTO (máx. 300 palabras)** |
| --- |
| Este proyecto se centrará en explorar respuestas a la visualización compasiva, una téncica de la terapia centrada en la compasión (Compassion Focused Therapy; CFT; p.ej. Gilbert, 2014). Existe fuerte evidencia que documenta que la CFT baja la depresión, vergüenza, la autocrítica, y aumenta el estado de ánimo (Kirby, 2016).  La CFI involucra visualizar compasión hacia los demás o imaginar personas, lugares u objetos dirigiendo compasión hacia uno mismo. Ensayos únicos de CFI han demostrado una reducción del afecto negativo y de los cambios fisiológicos asociados a la atenuación de los comportamientos centrados en amenazas (por ejemplo, Rockliff et al., 2008). Asimismo, se ha encontrado que la práctica regular de CFI aumenta la autocompasión y reduce el afecto negativo tanto en población clínica como no-clínica (Gilbert & Irons, 2004; McEwan & Gilbert, 2016). De igual manera, se identifica que la CFI puede crear respuestas centradas en amenazas en algunos individuos. No obstante, dichos hallazgos se han basado en tareas que involucran recibir compasión de los demás.  De ahí, el presente estudio tiene como objetivo explorar las respuestas de los participantes a ejercicios de visualización que involucran la autocompasión, en comparación con una tarea de relajación (con el fin de controlar ciertas demandas de la tarea excluyendo el componente de la compasión) y una tarea control (leer una revista). |
| **OBJETIVO GENERAL** |
| Explorar las respuestas de población clínica a la visualización autocompasiva en comparación con la relajación y una tarea control. |
| **OBJETIVOS ESPECÍFICOS** |
| 1. ¿Cómo responden los participantes clínicos inicialmente a la visualización autocompasiva en un ensayo inicial, en comparación con relajación y una tarea control (usando medidas de autoreporte y variabilidad del ritmo cardíaco)? 2. ¿Se reducen las respuestas de amenaza después de repetidos ensayos de visualización autocompasiva? |
| **METODOLOGÍA (tipo de estudio, instrumentos, lugar y personas 300 palabras)** |
| Participantes y proceso de reclutarlos:  Queremos reclutar una muestra de 25 estudiantes para este estudio, basado en otros estudios sobre visualización compasiva con un diseño similar (Rockliff et al., 2008; Duarte et al., 2015).  Los criterios de inclusión serán:  1.Nivel clínico de ansiedad o depresión (definido como ≥ 8 en el OASIS o ODSIS)  2. Nivel alto de auto-crítica (seleccionamos el punto de corte de 0,5 desviaciones estándar por encima de la media en auto-insuficiencia u odio a sí mismo en la FSCRS. Basado en una validación en Colombia de la FSCRS por Naismith, Duran Ferro, Ingram, & Jiménez Leal (Entregada), aceptaremos a los participantes que han puntuado ≥ 24 en auto-insuficiencia y ≥ 8 para odio a sí mismo.  Los criterios de inclusión fueron seleccionados ya que las intervenciones están diseñadas para ayudar una población clínica con alta auto-crítica. Ofrecerlo a una población no-clínica (i) reduce la posibilidad de observar cambios en los resultados, y (ii) no ayuda a las personas que presentan mayor necesidad.  No obstante, las personas que completen la evaluación inicial y no cumplan con los criterios de inclusión serán invitados a una sesión grupal para aprender técnicas de compasión. Además, recibirán materiales sobre la compasión por correo al final del estudio.  Al interior del campus universitario (Universidad de los Andes) se colgarán carteles publicitarios y el mismo formato será utilizado en las redes sociales.  Diseño:  Los participantes serán aleatorizados para completer 3 o 4 ensayos (ver detalles a continuación) usando una proporción de 2:1 y usando una secuencia de aleatorización elaborada antes del inicio del estudio. Esto nos permitirá explorar en un pequeño subgrupo si un cuarto ensayo impacta los resultados. Asimismo, estamos llevando a cabo un estudio relacionado, el cual explorará los efectos de la psicoterapia que se ofrecerá posteriormente a estos 3-4 ensayos. Anticipamos que un cuarto esayo aumentará la probabilidad de abandono del segundo estudio. Por lo tanto, no asignaremos a todos los participantes para que realicen el cuarto ensayo.  Instrumentos:  Por favor remitirse a Anexo 2 - cuestionarios, para copias de los cuestionarios que usaremos que nos son validados.   1. **Demográficos** 2. **Formato de auto-reporte - Variación fisiológica** 3. **Overall Depression Severity and Impairment Scale (ODSIS)** 4. **Overall Anxiety Severity and Impairment Scale (OASIS)** 5. **Forms of Self-Criticism/Attacking and Self-Reassuring Scale (FSCRS)** 6. **Afecto Positivo y Negativo Generado** 7. **Heart Rate Variability (HRV) - Variabilidad de Ritmo Cardiaco (VRC)**   La variabilidad de Ritmo Cardiaco (VRC) es una medida fisiológica que permite cuantificar pequeños cambios en ansiedad momento por momento, que un cuestionario no podría cuantificar. En este estudio, nos permitirá medir si los participantes responden a la compasión con ansiedad o con relajación (esperamos que después de la intervención, todos respondan con relajación, pero predecimos que al inicio algunas personas responderán con ansiedad).  Utilizaremos el sistema BioPac para tomar datos de VRC. Los 3 electrodos seguirán un sistema Lund de ubicación, ya que se consideran los más estables, poco invasivos y con mejor valor diagnóstico.  Utilizando el programa Acqknowledge, se analizarían las pulsaciones por minuto (BPM), y se analizarían los desequilibrios de la actividad simpática y parasimpática de la VRC. Específicamente, el dominio temporal que proporcionan información de la raíz cuadrada del valor medio de la suma de las diferencias al cuadrado de todos los intervalos RR sucesivos (RMSSD) (Task Force of the European Society of Cardiology and the North American Society of Pacing and Electrophysiology, 1996). RMSSD was selected because there is no agreed clinically-significant change value for anlysing HF HRV data (see Data analysis section).  Procedimiento:  Los participantes completarán un cuestionario de evaluación inicial en linea que incluirá: un consentimiento informado (ver el apéndice 1), preguntas demográficas, ODSIS, OASIS y FSCRS. Los participantes elegibles serán invitados a asistir a sesiones presenciales. En cada sesión, un asistente de investigación medirá la variabilidad del ritmo cardíaco (VRC) mientras el participante se involucra en tres tareas de 4 minutos de duración en el siguiente orden:   - **Tarea de control:** los participantes leerán una revista con contenido neutral durante 4 minutos. - **Visualización de relajación** (Ver Anexo 3)**:** los participantes realizarán un ejercicio de visualización de una playa o bosque durante 4 minutos. - **Visualización compasiva:** los participantes recibirán un ejercicio de visualización de 4 minutos (Ver Anexo 3).   **Data analysis**  Se ejecutarán pruebas t pareadas, (i) para estimar las diferencias en la VRC durante la visualización autocompasiva, la visualización de relajación y la tarea de control, (ii) para examinar los cambios en el afecto positivo y negativo autoreportados del pre- al post-CFI y (iii) para explorar si los cambios en el afecto positivo y negativo durante CFI son mayores o menores desde el ensayo 1 hasta el ensayo 3.  Con el fin de complementar los análisis a nivel grupal, utilizaremos análisis de cambios confiables y clínicamente significativos. En primer lugar, reportaremos cuántos participantes muestran un cambio confiable (Jacobson y Truax, 1991) en el afecto positivo o negativo después de la CFI.  También calcularemos cuántos individuos muestran una respuesta de VRC clínicamente significativa (≥5 ms de cambio en RMSSD dentro de un individuo entre dos tareas diferentes).  Por último, reportaremos el número de respuestas clínicamente significativas positivas y negativas para la relajación y la compasión en cada ensayo, con el fin de (i) comparar los efectos de la compasión frente a la relajación en el ensayo 1 y (ii) explorar si los ensayos repetidos mejoraran la respuesta a cada tarea. |
| **CONSIDERACIONES ÉTICAS (máx. 300 palabras)** |
| **Consideraciones éticas**  Se seguirán los principios éticos de investigación en Psicología planteados por la American Psychological Association para minimizar posibles riesgos para las participantes. A continuación se explican estos principios:  **Respeto por las personas:**  Este principio contempla dos aspectos que son la autonomía y la protección de las personas vulnerables. Con el fin de asegurar el tratamiento respetuoso y garantizar la seguridad, se desarrollarán las siguientes acciones:  a) se garantizará que nadie más que el asistente de investigación y su supervisora (Dr Iona Naismith) puedan tener acceso a conectar las respuestas a los cuestionarios con los detalles identificables de cada participante;  b) las respuestas a los cuestionarios serán codificadas, guardadas y aseguradas en la oficina asegurada de la investigadora principal;  c) se ofrecerá información general de los resultados a quienes lo soliciten.  Aunque las sesiones no son terapia y no involucran una conversación sobre la condición del participante, es posible que ellos reporten algo problemática en la sesión (ej. intento suicidio, autolesión). Si esto pasa, los participantes serán remitidos al servicio de crisis ofrecido por la Casa Espinosa. La supervisora confirmará que los facilitadores de las sesiones conozcan el proceso antes del comienzo del estudio.  El consentimiento informado también tiene información sobre lugares en que los participantes puedan acceder a ayuda profesional en un crisis.  **Minimizar los riesgos para las participantes:**  Las sesiones se llevarán a cabo en el consultorio de la Universidad que tiene el sistema BioPac. Aunque la sesión no es de psicoterapia formal, hemos determinado que el consultorio es a prueba de sonido, lo cual garantiza la confidencialidad de los participantes.  A las participantes se les permitirá retirarse del estudio, si así lo desean. Esto está incluido en el consentimiento informado.  Las sesiones serán facilitadas con grabaciones para estandarizar el contenido y mantener alta calidad. Serán facilitados por un estudiante de la maestría clínica de psicología o un estudiante de psicología en práctica. |
| **Posibles beneficios:**   1. Practicar dos técnicas implementadas en la Terapia Centrada en la Compasión (Visualización Compasiva y Relajación).   **Principio de igualdad**  Con el fin de brindar a aquellos participantes que, dados sus puntajes en los cuestionarios de auto-reporte, no logran ser parte de la muestra clínica objeto del presente estudio, la oportunidad de obtener los beneficios, se realizarán procesos grupales. Durante estos procesos, se manejarán distintos horarios (suficientemente flexibles para que todos puedan asistir), dentro de los cuales se dé una charla sobre la compasión y una oportunidad de practicar la visualización compasiva. |

# **Referencias**

Ascone, L., Sundag, J., Schlier, B., & Lincoln, T. M. (2017). Feasibility and effects of a brief compassion-focused imagery intervention in psychotic patients with paranoid ideation: A randomized experimental pilot study. Clinical Psychology & Psychotherapy, 24, 348-358. https://doi.org/10.1002/cpp.2003

Bentley, K.H., Gallagher, M.W., Carl, J.R. & Barlow, D.H. (2014). Development and validation of the Overall Depression Severity and Impairment Scale. *Psychol Assess, 26*(3), 815–30. <http://doi.apa.org/getdoi.cfm?doi=10.1037/a0036216>

Brennan, M., Palaniswami, M., Kamen P. (2001). Do existing measures of Poincare plot geometry reflect nonlinear features of heart rate variability? *IEEE Trans Biomed Eng. 48*(7). P.1342.

Gilbert, P. (2014). Terapia centrada en la compasión: características distintivas. Spain: Desclée de Brouwer.

Holmes, E. A., & Mathews, A. (2010). Mental imagery in emotion and emotional disorders. *Clinical Psychology Review, 30*(3), 349–362.

Jacobson N, Truax PN. (1991). Clinical significance: a statistical approach to defining meaningful change in psychotherapy research. *Journal of Consulting and Clinical psychology, 59*(1), 12–19. <https://doi.org/10.1037//0022-006x.59.1.12>.

Kirby, J. (2016). Compassion interventions: The programmes, the evidence, and implications for research and practice. *Psychology and Psychotherapy: theory, research and practice, 90* (3), 432-455.

Lincoln, T. M., Hohenhaus, F., & Hartmann, M. (2013). Can paranoid thoughts be reduced by targeting negative emotions and self-esteem? An experimental investigation of a brief compassion-focused intervention. *Cognitive Therapy and Research, 37*(2), 390–402. <https://doi.org/10.1007/s10608-012-9470-7>.

McEwan, K. & Gilbert, P. (2016).A pilot feasibility study exploring the practising of compassionate imagery exercises in a nonclinical population. *Psychological Psychotherapy: Theory, Research & Practice, 89(2*):239–43. Available from: <http://doi.wiley.com/10.1111/papt.12078>

Naismith, I., Mwale, A., Feigenbaum, J. (2018). Inhibitors and facilitators of Compassion-Focused Imagery in Personality Disorder. Clinical Psychology & Psychotherapy. Advanced online publication. https://doi: 10.1002/cpp.2161.

Norman, S.B., Hami Cissell, S., Means-Christensen, A.J., Stein, M.B. (2006). Development and validation of an Overall Anxiety Severity and Impairment Scale (OASIS). *Depression & Anxiety, 23*(4):245–9. http://doi.wiley.com/10.1002/da.20182

Task Force of the European Society of Cardiology and the North American Society of Pacing and Electrophysiology. (1996). Heart Rate Variability. Standards of measurement, physiological interpretation, and clinical use. *Eur Heart J, 17*(81). P, 354.

Rockliff, H., Gilbert, P., McEwan, K., Lightman, S., Glover, D. (2008). A pilot exploration of heart rate variability and salivary cortisol responses to compassion-focused imagery. *Clinical Neuropsychiatry: Journal of Treatment Evaluation, 5*(3), 132–139.

**ANEXO 1 - Consentimiento informado.**

**(Nota: este es el consentimiento que se utilizará para el estudio más grande que involucra las sesiones de psicoterapia, además de las medidas tomadas para este estudio).**


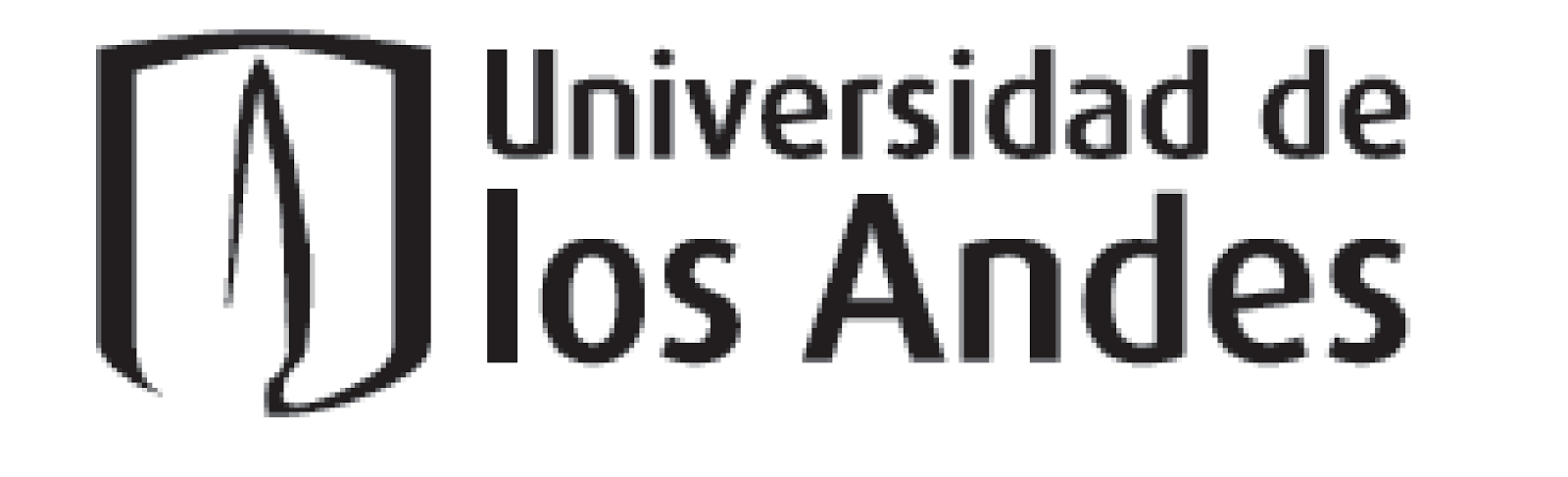


**UNIVERSIDAD DE LOS ANDES**

Facultad de Ciencias Sociales

Departamento de Psicología

Miembro de la Asociación Colombiana de Facultades de Psicología

**Estudio: Desarrollar el bienestar con ejercicios de la compasión**

Queremos invitarlo a participar en un proyecto de investigación que tiene como objetivo evaluar una intervención de bienestar centrada en la auto-compasión. El programa busca aumentar el bienestar emocional y disminuir emociones y pensamientos que interfieren con su funcionamiento en diferentes áreas de su vida.

Su participación en esta investigación será muy importante porque a partir de los datos recopilados esperamos identificar los componentes más eficaces del programa y así será posible ayudar a otras personas. Este proyecto cuenta con el aval del Comité de Ética de la Investigación de la Universidad de los Andes.

Su participación en este proyecto consistirá en:

(i) completar una evaluación en línea hoy (15-20 minutos) y otra en una semana (5 minutos); (ii) asistir a siete sesiones individuales de 40-45 minutos cada una, en las que aprenderá técnicas para incrementar el bienestar y manejar la tristeza o preocupación; (iii) una evaluación en línea 1 mes después (5 minutos).

Nos interesa conocer las experiencias de los participantes en el programa, con el fin de mejorarlo continuamente. Con este objetivo, todas las sesiones individuales serán grabadas en audio. La información que se recoge en este estudio a través de cuestionarios y grabaciones de audio es estrictamente confidencial y será utilizada exclusivamente para fines investigativos; en ningún momento se dará a conocer su identidad. Los cuestionarios serán identificados con un código específico, el cual será utilizado durante el procesamiento y análisis de los datos. Todos los materiales del presente estudio se mantendrán archivados bajo llave en la oficina del investigador en la Universidad de los Andes, y los archivos digitales estarán protegidos con contraseñas que solamente conocerá el equipo de investigación. Al finalizar el estudio se entregará un resumen con los resultados principales a los participantes que lo soliciten.

Su participación en esta investigación no tiene ningún riesgo psicológico o físico. Sin embargo, si alguna pregunta del cuestionario le llegase a generar inquietud o incomodidad, usted podrá omitir la pregunta. Su participación en esta investigación es totalmente voluntaria y está en completa libertad de retirarse en el momento que lo desee, sin que esto tenga repercusiones de ninguna naturaleza. Si su nivel de incomodidad fuese alto, los asistentes de investigación le proporcionarán información sobre servicios de atención psicológica disponibles en la ciudad.

En este estudio usted aprenderá técnicas eficaces de regulación emocional. Sin embargo, no constituye psicoterapia (que es adaptada específicamente para los problemas que quieras resolver). Para obtener esto (por un costo), por favor contactar al Servicio de Atención Psicológica, Universidad de los Andes (<https://decanaturadeestudiantes.edu.co/index.php/es/consejeria-academica-y-vocacional/consejeria-psicologica>).

En caso de que usted tenga alguna inquietud sobre esta investigación puede comunicarse con la profesora Iona Naismith de la Universidad de los Andes al número telefónico 3324365, o al correo electrónico ij.naismith@uniandes.edu.co. Si tuviese inquietudes sobre aspectos éticos de esta investigación puede contactar al Comité de Ética de la Universidad de los Andes a través del correo electrónico comité[-etica-investigaciones@uniandes.edu.co](mailto:-etica-investigaciones@uniandes.edu.co).

Muchas gracias por su colaboración

Cordialmente,

_____________________

Iona Naismith

Profesora Asistente

Departamento de Psicología

Universidad de los Andes

Conozco el propósito de la investigación:  ***Desarrollar el bienestar con ejercicios de la compasión***  y lo que implica mi participación en la misma. Entiendo que mi participación es voluntaria y que puedo retirarme en cualquier momento. También entiendo que, si deseo recibir un informe sobre los resultados del estudio, este será enviado al correo electrónico que registro a continuación.

Nombre _______________________________________________________________

Firma _________________________________________________________________

Cédula __________________________________ Fecha _______________________

Correo electrónico _____________________

**Investigador:**

Nombre _______________________________________________________________

Firma _________________________________________________________________

Cédula __________________________________ Fecha _______________________

**Testigo 1**

Nombre _______________________________________________________________

Firma _________________________________________________________________

Cédula ________________________________ Fecha _________________________

**Testigo 2**

Nombre _______________________________________________________________

Firma _________________________________________________________________

Cédula ________________________________ Fecha _________________________

**ANEXO 2 - Cuestionarios.**

**Demográficos**

A través de la plataforma en línea:

Edad (años) ___________

Sexo: Mujer __ Hombre__ Prefiero no especificar __

Estado civil: Soltero/a ___Casado/a__ Divorciado/a__ Viudo/a

**Formato de auto-reporte - variación fisiológica**

**Descripción:** Ya que la Variabilidad del Ritmo Cardiaco resulta crucial para el presente estudio, al inicio de cada sesión se presentará un formato breve indagando algunos datos posibles que podrían incidir en esta medida fisiológica.

Dentro de las medidas tomadas para el presente estudio, se encuentran aquellas fisiológicas que podrían cambiar según diversos factores. Para nosotros es importante lograr una medición precisa y reconocer aquellos casos en que estas mediciones fisiológicas puedan verse alteradas.

Por favor, marque con una X según sea el caso y especifique de ser necesario.

En las últimas 2 horas, usted ha:

___ Hecho ejercicio prolongado _______________________________________

___ Comido _______________________________________________________

___ Bebido ________________________________________________________

A partir de la semana anterior ¿Ha habido algún cambio en sus medicamentos?

___ Inicio _________________________________________________________

___ Finalización ____________________________________________________

___ Cambio de dosis _________________________________________________

***Escala de Afecto Positivo y Negativo Generado***

***Califique sus emociones durante la visualización, utilizando la siguiente escala:***

***
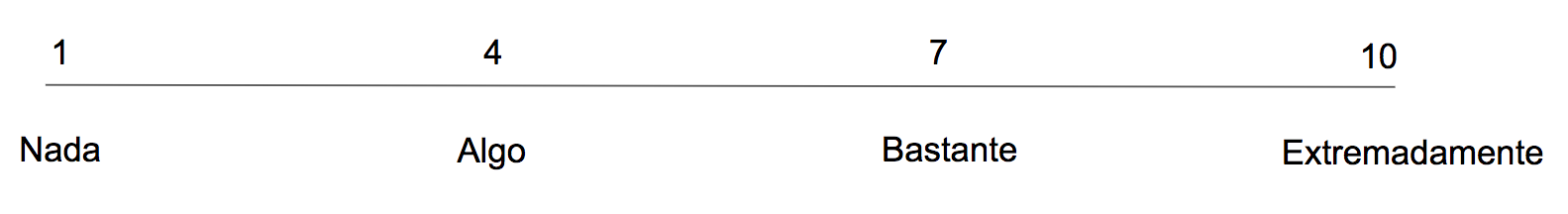
***

|  | **1** | **2** | **3** | **4** | **5** | **6** | **7** | **8** | **9** | **10** |
| --- | --- | --- | --- | --- | --- | --- | --- | --- | --- | --- |
| **Me sentí tranquilo(a)** |  |  |  |  |  |  |  |  |  |  |
| **Me sentí relajado(a)** |  |  |  |  |  |  |  |  |  |  |
| **Me sentí seguro(a)** |  |  |  |  |  |  |  |  |  |  |
| **Me sentí contento(a)** |  |  |  |  |  |  |  |  |  |  |
| **Me sentí ansioso/a** |  |  |  |  |  |  |  |  |  |  |
| **Me sentí angustiado/a** |  |  |  |  |  |  |  |  |  |  |
| **Me sentí vulnerable** |  |  |  |  |  |  |  |  |  |  |
| **Me sentí inseguro** |  |  |  |  |  |  |  |  |  |  |

**ANEXO 3 - Guiones**

***Nota:** El guión será grabado en audio y reproducido según el cronograma de las sesiones.

**GUIÓN DE VISUALIZACIÓN COMPASIVA (Gilbert).**

Actuando hacia nosotros de una manera afectuosa, amable y compasiva es una manera de manejar las emociones difíciles. Intentaremos desarrollar varias características compasivas en este ejercicio. No importa si piensas que tienes estas características o no; lo importante es imaginar que las tienes. Por favor, siéntate en una posición cómoda. Deja que tus ojos se cierren, total o parcialmente. Respira profundamente para acomodarte a tu cuerpo y al momento presente. Pon tu mano en el pecho, sobre tu corazón, o donde sea cómodo. Con amabilidad enfoca tu atención en ti mismo. Ahora hazte consciente de tu respiración e intenta respirar lentamente. Siente como tu cuerpo inhala y exhala, y si tu atención se dispersa, vuelve a enfocarte en tu respiración otra vez.

Primero, imagínate que eres una persona muy muy sabia. Sabes que la vida puede ser difícil sin que esto sea tu culpa, todos cometemos errores. Si cometes un error, te motivas a cambiar de manera amable, y no a través de culparte o criticarte sobre acciones que ya hiciste.

Segundo, imagínate que tienes la fuerza para tolerar tus dificultades y emociones, y la confianza de aceptarlos sin juzgarte.

Tercero, imagínate que tienes una manera muy cálida y afectuosa de tratarte a ti mismo. Imagínate hablándote con cálidad y amabilidad. Intenta tener una expresión facial amable mientras lo haces, tal vez con una sonrisa amable.

Finalmente, te comprometes a estar ahí para ti, a ser tu apoyo. Aún en las situaciones más dificiles te comprometes a no juzgarte y no a culparte por tus errores. Imagínate siendo tu propio apoyo, y estar ahí para ti. Aún en las situaciones más difíciles, te comprometes a no juzgarte y no ha culparte por tus errores, en cambio e ayudas y te das lo que necesitas.

Sigue respirando lentamente. Repite en tu mente las siguientes palabras de compasión, de manera amable y cálida, sintiendo su importancia.

o Quiero que seas feliz. (Usar tu nombre podría facilitar el ejercicio)

o Quiero que estés en paz.

o Quiero que tengas salud y bienestar.

o Quiero que vivas tranquilamente.

Por último, respira varias veces profundamente y simplemente trata de disfrutar cualquier sentimiento de tranquilidad que pueda surgir. Lentamente, abre tus ojos.

**Visualización relajante 1 - Playa**

En este ejercicio de imaginación vamos a crear una imagen de nosotros mismos haciendo algo relajante.

Coloca ambos pies sobre el suelo, con los hombros separados y extendidos, y descansa las manos sobre las piernas. Cierra los ojos o mira al suelo si lo prefieres.

Gentilmente, concéntrate en tu respiración. Respira desde tu abdomen algunas veces. Nota el flujo de aire entrando y saliendo por tu nariz. No necesitas cambiar nada, simplemente permite que las cosas sean como son.

Está bien que tu mente divague – cuando esto suceda simplemente nótalo con curiosidad y, gentilmente, guía tu mente de vuelta a la respiración.

Siente tus brazos y tus piernas soltándose y relajándose… y tus hombros también…

Cuando estés listo, imagina que estás caminando por un largo tramo de arena blanca, hacia el océano… Estas mirando hacia el agua… es de color azul y verde.

Puedes escuchar las olas más adelante… puedes oler la brisa del mar… sientes una placentera y fría brisa fluyendo…

La arena es caliente, y muy suave. Imagínate quitándote los zapatos y caminando por la playa.

Escucha las olas chocando en la costa… Siente el claro olor a sal del agua.

Permítete sentirte contenido y relajado, permite que estos sentimientos crezcan. Recuerda mantener tu postura corporal tan relajada como puedas.

Pasea por la playa, por el borde del agua… libera tus preocupaciones… cálmate… disfruta el momento…

Más adelante hay una cómoda silla con una toalla, sólo para ti…

Siéntate o recuéstate en la silla, o extiende la toalla en la arena… relájate ahí… disfruta el sol… la briza… las olas…

Te sientes tranquilo y relajado…

Cuando te sientas listo para regresar de ese lugar tranquilo, lentamente regresa a tu nivel usual de alerta y conciencia…Gentilmente, deja que la imagen se desvanezca, y sal del ejercicio, volviendo a la habitación.

**Visualización relajante 2 - Bosque**

En este ejercicio de imaginación vamos a crear una imagen de nosotros haciendo algo relajante.

Coloca ambos pies planos sobre el suelo, con el ancho de tus hombros separados, y descansa las manos sobre las piernas. Cierra los ojos o mira al suelo si lo prefieres.

Gentilmente, concéntrate en tu respiración. Respira desde tu abdomen algunas veces. Nota el flujo de aire entrando y saliendo por tu nariz. No necesitas cambiar nada, simplemente permite que las cosas sean como son.

Está bien que tu mente divague – cuando esto suceda simplemente nótalo con curiosidad y, gentilmente, guía tu mente de vuelta a la respiración.

Siente tus brazos y tus piernas soltándose y relajándose… y tus hombros también…

Cuando estés listo, imagínate que estás caminando por un camino en medio del bosque. El camino es suave debajo de tus zapatos, es una mezcla de tierra, hojas caídas, y musgo. A medida que caminas, tu cuerpo se relaja y tu mente se aclara, más y más con cada paso.

Respira el aire fresco de la montaña, llena tus pulmones completamente. Ahora exhala, suelta todo el aire. Siente renovado.

El aire es fresco, pero agradable. El sol se filtra por los árboles, haciendo un patrón moteado, que se mueve en el suelo delante de ti.

Escucha los sonidos del bosque… los pájaros cantan. Una brisa gentil sopla. Las hojas en los árboles cambian y se balancean con el viento suave.

Mientras caminas por el bosque, siente que tus músculos se relajan y se alargan. Mientras tus brazos se balancean al ritmo de tu caminar, se vuelven sueltos, relajados y flojos.

Tus piernas y parte inferior del cuerpo también se relajan, se sienten libres y relajados. Siente la tensión que abandona tu cuerpo mientras admiras el paisaje que te rodea.

Más adelante hay una roca grande y lisa, bajo el sol... como una silla esperando que descanse. Siéntate o acuéstate sobre la roca si lo deseas. Es muy cómoda. Te sientes muy cómodo y a gusto. El sol brilla sobre ti. Disfruta de este lugar tranquilo por unos momentos.

Cuando estés listo para salir de este lugar pacífico, comienza lentamente a volver a despertar tu cuerpo. Gentilmente, deja que la imagen se desvanezca, y sal del ejercicio, volviendo a esta habitación.
