# Supplementary material for: Physiological response to self-compassion versus relaxation in a clinical population
Source: PLoS One. 2023 Feb 7;18(2):e0272198. doi: 10.1371/journal.pone.0272198 (PMC9904495; doi:10.1371/journal.pone.0272198)
Supplement: S2 File — (DOCX) [file pone.0272198.s004.docx]

**Presentation of Projects to the Ethics Committee:**

**Faculty of Social Sciences**

| **Project Title: Exploration of responses to compassion in a clinical population** |
| --- |
| **Department: Psychology** |
| **Researcher: Iona Naismith** |
| **Anticipated research start date: 15 march, 2018** |

| **Project executive summary (maximum 300 words)** |
| --- |
| This project focuses on exploring responses to compassionate imagery, a technique from  Compassion Focused Therapy (CFT; Gilbert, 2014). Evidence has documented that CFT decreases depression, shame, self-criticism and increases self-esteem (Kirby, 2016).  Compassion-focused imagery (CFI) is a key technique in CFT, which involves visualizing compassion towards others, or imagining people, places or objects directing compassion towards oneself. Single trials of CFI have shown a reduction of negative affect and physiological changes associated with the attenuation of threat-focused behaviors (e.g. Rockliff et al., 2008). Regular CFI practice has increased self-compassion and reduced negative affect in both clinical and non-clinical populations (Gilbert & Irons, 2004; McEwan & Gilbert, 2016). Unfortunately, CFI can create threat-focused responses in some individuals. However, these findings have been based on tasks involving receiving compassion from others.  The present study therefore aimed to explore participants’ responses to imagery exercises involving self-compassion, in comparison to a relaxation task (to control for certain task demands but without the compassion element), and a control task ( reading a magazine). |
| **GENERAL OBJECTIVE** |
| To explore responses to self-compassionate imagery in comparison to relaxation and a control task, in a clinical population. |
| **SPECIFIC OBJECTIVES** |
| 1. How do clinical participants initially respond to *self*-compassionate imagery in an initial trial, compared to relaxation and a control task? (using self-report and HRV measures) 2. Do any threat responses reduce following repeated trials of self-compassionate imagery? |
| **METHODS (maximum 300 words)** |
| Participants and process of recruitment:  We plan to recruit a sample of 25 participants for this study, based upon other studies of compassionate imagery with similar designs (Rockliff et al., 2008; Duarte et al., 2015).  Inclusion criteria are:  1.Clinical level of anxiety or depression (defined for this study ≥ 8 on the OASIS or ODSIS)  2. High self-criticism (we selected a cut-off of 0.5 standard deviations above the mean in self-inadequacy or self-hatred on the FSCRS). Based upon a validation in Colombia of the FSCRS by Naismith, Duran Ferro, Ingram, & Jiménez Leal (Submitted), this represents ≥ 24 in self-inadequacy or ≥ 8 in self-hatred.  These criteria were selected because these interventions are designed to help a clinical population presenting with high self-criticism. Offering this to a non-clinical population (i) reduces the likelihood of observing significant changes, and (ii) will not allow us to help those who present with higher needs.  Nonetheless, those who complete the initial screening and do not meet inclusion criteria will be invited to a group session to learn compassion techniques. They will also receive compassion materials via email at the end of the study.  For recruitment, posters will be hung inside the campus of the University of the Andes. The same information will be published in university social media pages.  Design:  Participants will be randomized to complete 3 or 4 trials (see details below) using a 2:1 ratio, using a randomization sequence drawn up prior to the study start. This will allow us to explore in a small subgroup whether a fourth trial impacts results. We are also running a related study, which will explore the effects of psychotherapy that will be offered following these 3-4 trials, and we anticipate that a fourth trial will increase probability of dropout from the second study, therefore we will not assign all participants to do the fourth trial.  Measures:  See Appendix 2 for copies of the questionnaires that we intend to use that are not validated.   1. **Demographics** 2. **Self-report form – Physiological variation** 3. **Overall Depression Severity and Impairment Scale (ODSIS)** 4. **Overall Anxiety Severity and Impairment Scale (OASIS)** 5. **Forms of Self-Criticism/Attacking and Self-Reassuring Scale (FSCRS)** 6. **Positive and negative affect generated** 7. **Heart Rate Variability (HRV).** Heart rate variability (HRV) is a physiological measure that allows us to quantify small changes in anxiety moment by moment, that a questionnaire cannot quantify. In this study it will allow us to measure whether participants respond to compassion with a threat-based response or a relaxed response (we expect that after 3-4 trials, all participants will respond with relaxation or a neutral response, but we predict that at the start, some will respond with threat-based responses).   We will use the BioPac system to collect HRV data. Electrodes will be placed using the Lund guidance which is considered the most stable, least invasive, and with high diagnostic accuracy. Using the program Acqknowledge, we will analyse beats per minute (BPM) and ratios of sympathetic and parasympathic activity. Specifically, we will use the Root Mean Square of the Successive Differences (RMSSD) of RR intervals (Task Force of the European Society of Cardiology and the North American Society of Pacing and Electrophysiology, 1996). RMSSD was selected because there is no agreed clinically-significant change value for anlysing HF HRV data (see Data analysis section).  Procedure  Participants will complete an initial screening questionnaire online including: an informed consent (see Appendix 1), demographics questions, ODSIS, OASIS and FSCRS. Eligible participants will be invited to attend in-person sessions. In each session, HRV will be measured by a research assistant whilst the participant engages in three 4-minute tasks in the following order:   - **Control task:** participants will read a magazine with neutral content for 4 minutes - **Relaxation imagery** (see appendix 3): participants will complete a relaxation exercise of beach or forest imagery for 4 minutes. - **Compassion-focused imagery:** participants will complete a compassion-focused imagery for 4 minutes (see appendix 3).   **Data analysis**  Paired *t-*tests will be run (i) to estimate differences in HRV during the self-compassion imagery, relaxation imagery and the control task, (ii) to examine changes in self-reported positive and negative affect from pre-CFI to post-CFI, (iii) to explore whether changes in positive and negative affect during CFI are greater or smaller from Trial 1 to Trial 3.  To complement group-level analyses, we will use reliable and clinically-significant change analyses. We will firstly report how many participants show a reliable change (Jacobson & Truax, 1991) in positive or negative affect following CFI.  We will also calculate how many individuals show a clinically-significant HRV response (≥ 5ms change in RMSSD within one individual between two different tasks).  Finally, we will report the number of positive and negative clinically-significant responses for both relaxation and compassion in each trial, in order to (i) compare effects of compassion versus relaxation at trial 1, and (ii) explore whether repeated trials improved response to each task. |
| **Ethical considerations (máx. 300 words)** |
| **Ethical consideration**  This study will follow the ethical principles in psychology proposed by the American Psychological Association to minimize possible risk to participant. These principles are elaborated below:  **Respect for participants:**  This prinicple covers two aspects, autonomy and protection of vulnerable people. With the aim of assuring respectful treatment and guarantee of safety, the following actions will be taken:   1. Research team will guarantee that only the research assistant(s) and supervisor (Dr Iona Naismith) will have access to the database which connects the questionnaire responses with the identifiable details of each participant, 2. Responses to questionnaires will be encrypted and secured in the office of the principle investigator, 3. General information (anonymized) of the study results will be sent to participants who request it.   Although these sessions are not therapy and do not involve discussion of the participant’s functioning, it is possible that they report a problem in the session (e.g. suicidal ideation, self-harm). If this occurs, the participant will be referred to the crisis service of Casa Espinosa. The supervisor will confirm that the session facilitators are aware of this procedure before the study begins. The informed consent also contains information on places where participants can access professional help in a crisis.  **Minimizing risk for participants:**  Sessions will take place in an office in the university which contains the BioPac system. Although the sesión does not involve formal psychotherapy we have determined that the room is soundproof to guarantee participant confidentiality. Participants are able to withdraw from the study at any point, as outlined in the informed consent.  Sessions will be facilitated with audio recordings to standardize content and ensure high quality. They will be facilitated by a student of the Masters in clinical psychology or a student in an undergraduate research placement. |
| **Posible benefits**   1. Practice two techniques from Compassion focused therapy (compassionate imagery and relaxation).   **Principle of Equality**  With the aim of extending the study benefits to those participants who are not eligible to participate in the study due to their screening responses, they will be invited to two group sessions. These sessions will be offered at different times (sufficiently flexible so that all can attend), within which a talk on compassion will be given and participants will be invited to try out compassionate imagery. |

# **References**

Ascone, L., Sundag, J., Schlier, B., & Lincoln, T. M. (2017). Feasibility and effects of a brief compassion-focused imagery intervention in psychotic patients with paranoid ideation: A randomized experimental pilot study. Clinical Psychology & Psychotherapy, 24, 348-358. https://doi.org/10.1002/cpp.2003

Bentley, K.H., Gallagher, M.W., Carl, J.R. & Barlow, D.H. (2014). Development and validation of the Overall Depression Severity and Impairment Scale. *Psychol Assess, 26*(3), 815–30. <http://doi.apa.org/getdoi.cfm?doi=10.1037/a0036216>

Brennan, M., Palaniswami, M., Kamen P. (2001). Do existing measures of Poincare plot geometry reflect nonlinear features of heart rate variability? *IEEE Trans Biomed Eng. 48*(7). P.1342.

Gilbert, P. (2014). Terapia centrada en la compasión: características distintivas. Spain: Desclée de Brouwer.

Holmes, E. A., & Mathews, A. (2010). Mental imagery in emotion and emotional disorders. *Clinical Psychology Review, 30*(3), 349–362.

Jacobson N, Truax PN. (1991). Clinical significance: a statistical approach to defining meaningful change in psychotherapy research. *Journal of Consulting and Clinical psychology, 59*(1), 12–19. <https://doi.org/10.1037//0022-006x.59.1.12>.

Kirby, J. (2016). Compassion interventions: The programmes, the evidence, and implications for research and practice. *Psychology and Psychotherapy: theory, research and practice, 90* (3), 432-455.

Lincoln, T. M., Hohenhaus, F., & Hartmann, M. (2013). Can paranoid thoughts be reduced by targeting negative emotions and self-esteem? An experimental investigation of a brief compassion-focused intervention. *Cognitive Therapy and Research, 37*(2), 390–402. <https://doi.org/10.1007/s10608-012-9470-7>.

McEwan, K. & Gilbert, P. (2016).A pilot feasibility study exploring the practising of compassionate imagery exercises in a nonclinical population. *Psychological Psychotherapy: Theory, Research & Practice, 89(2*):239–43. Available from: <http://doi.wiley.com/10.1111/papt.12078>

Naismith, I., Mwale, A., Feigenbaum, J. (2018). Inhibitors and facilitators of Compassion-Focused Imagery in Personality Disorder. Clinical Psychology & Psychotherapy. Advanced online publication. https://doi: 10.1002/cpp.2161.

Norman, S.B., Hami Cissell, S., Means-Christensen, A.J., Stein, M.B. (2006). Development and validation of an Overall Anxiety Severity and Impairment Scale (OASIS). *Depression & Anxiety, 23*(4):245–9. http://doi.wiley.com/10.1002/da.20182

Task Force of the European Society of Cardiology and the North American Society of Pacing and Electrophysiology. (1996). Heart Rate Variability. Standards of measurement, physiological interpretation, and clinical use. *Eur Heart J, 17*(81). P, 354.

Rockliff, H., Gilbert, P., McEwan, K., Lightman, S., Glover, D. (2008). A pilot exploration of heart rate variability and salivary cortisol responses to compassion-focused imagery. *Clinical Neuropsychiatry: Journal of Treatment Evaluation, 5*(3), 132–139.

**Appendix 1 – Informed consent**

**(Note: This is the same consent form that will be used in the larger study mentioned above, which involves sessions of psychotherapy along with the measures taken for the present study).**

UNIVERSITY OF THE ANDES

Faculty of Social Sciences

Department of Psychology

**Research Study: Developing well-being with compassion exercises**

We would like to invite you to participate in a research project that aims to evaluate a wellness intervention focused on self-compassion. The program seeks to increase emotional well-being and decrease emotions and thoughts that interfere with functioning in different areas of your life.

Your participation in this research is important because from the data collected we hope to identify the most effective components of the program and thus be able to help others. This project has the endorsement of the Research Ethics Committee of the University of the Andes.

Your participation in this project will consist of:

(i) completing an online assessment today (15-20 minutes) and another in a week (5 minutes); (ii) attending seven individual sessions of 40-45 minutes each, in which you will learn techniques to increase well-being and manage sadness or anxiety; (iii) an online evaluation 1 month later (5 minutes).

We are interested in knowing the experiences of the participants of the program in order to improve it. To this end, all individual sessions will be audio recorded. The information collected in this study through questionnaires and audio recordings is strictly confidential and will be used exclusively for research purposes; At no time will your identity be revealed. The questionnaires will be identified with a specific code, which will be used during data processing and analysis. All the materials of this study will be kept under lock and key in the researcher's office at the Universidad de los Andes, and the digital files will be protected with passwords that only the research team will know. At the end of the study, a summary with the main results will be delivered to the participants who request it.

Your participation in this research does not carry any psychological or physical risk. However, if any question on the questionnaire causes you concern or discomfort, you may skip the question. Your participation in this research is completely voluntary and you are completely free to withdraw at any time you wish without any repercussions. If your level of discomfort is high, the research assistants will provide you with information on psychological services available in the city.

In this study you will learn effective emotional regulation techniques. However, this program does not constitute psychotherapy, which is tailored specifically to the problems you want to solve. To obtain this (for a fee), please contact the Psychological Services of the Universidad de los Andes (https://decanaturadeestudiantes.edu.co/index.php/es/consejeria-academica-y-vocacional/consejeria-psicologica ).

If you have any concerns about this research, you can contact Professor Iona Naismith of the Universidad de los Andes at telephone number 3324365, or by email at ij.naismith@uniandes.edu.co. If you have concerns about the ethical aspects of this research, you can contact the Ethics Committee of the Universidad de los Andes through the e-mail address Comité-etica-investigaciones@uniandes.edu.co.

Thank you very much for your help

Cordially,

_____________________

Iona Naismith

Assistant Professor

Department of Psychology

University of the Andes

I know the purpose of the research study, “Developing well-being with exercises of compassion”, and what my participation in it implies. I understand that my participation is voluntary and that I can withdraw at any time. I also understand that if I wish to receive a report on the results of the study, it will be sent to the email address I register below.

Name _______________________________________________________________

Signature _________________________________________________________________

ID __________________________________ Date _______________________

Email _____________________

**Researcher:**

Name _______________________________________________________________

Signature _________________________________________________________________

ID __________________________________ Date _______________________

Email _____________________

**Witness 1**

Name _______________________________________________________________

Signature _________________________________________________________________

ID __________________________________ Date _______________________

Email _____________________

**Witness 2**

Name _______________________________________________________________

Signature _________________________________________________________________

ID __________________________________ Date _______________________

Email _____________________

**Appendix 2 – Questionnaires**

**Demographics**

Collected online:

Age (years) ___________

Sex: Female __ Male__ Prefer not to say __

Status: Single ___ Married__ Divorced__ Widowed____

**Self report form – Physiological variation**

**Description:** Since Heart Rate Variability is crucial for this study, a brief form will be presented at the beginning of each session, inquiring about criteria that could affect this measure. It is important for us to achieve an accurate measurement and to recognize those cases in which these physiological measurements may be altered.

Please mark with an X as appropriate and specify if necessary.

In the last 2 hours, have you:

___ Exercised for a long time _______________________________________

___ Eaten _______________________________________________________

___ Drunk ________________________________________________________

Since the previous week, have there been any changes in your medications?

___ Start _________________________________________________________

___ Completion ____________________________________________________

___ Dose change _______________________________________________

***Scale of Positive and Negative Affect Generated***

***Rate your emotions during the visualization, using the following scale:***

***None Some Fairly Extremely***

***
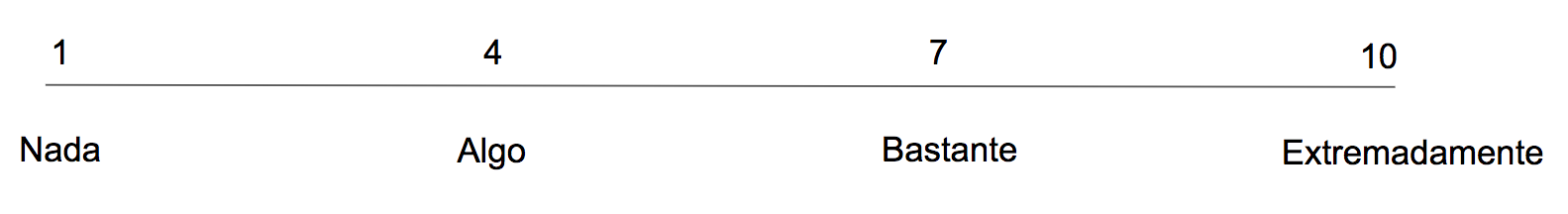
***

|  | **1** | **2** | **3** | **4** | **5** | **6** | **7** | **8** | **9** | **10** |
| --- | --- | --- | --- | --- | --- | --- | --- | --- | --- | --- |
| **I felt calm** |  |  |  |  |  |  |  |  |  |  |
| **I felt relaxed** |  |  |  |  |  |  |  |  |  |  |
| **I felt safe** |  |  |  |  |  |  |  |  |  |  |
| **I felt content** |  |  |  |  |  |  |  |  |  |  |
| **I felt anxious** |  |  |  |  |  |  |  |  |  |  |
| **I felt distressed** |  |  |  |  |  |  |  |  |  |  |
| **I felt vulnerable** |  |  |  |  |  |  |  |  |  |  |
| **I felt insecure** |  |  |  |  |  |  |  |  |  |  |

**Appendix 3 – Scripts for study**

*Script for self-compassionate imagery task (English translation)*

Acting towards ourselves in an affectionate, kind and compassionate manner is a way of managing difficult emotions. In this exercise, we will try to develop several characteristics of compassion. It doesn't matter if you think you have these characteristics or not; the important thing is to imagine that you do. Please sit in a comfortable position. Let your eyes close, fully or partially. Take a few deep breaths to settle into your body and into the present moment. Put your hand over your chest, heart, or wherever it feels comforting. Gently bring your awareness onto yourself. Now become aware of your breath and try to breathe slowly. Feel yourself breathing in and out. When your attention wanders, gently focus on your breath again.

First, imagine that you are a very, very wise person. You know that life can be difficult and that this is not your fault. We all make mistakes. Remember that when we make a mistake, the most helpful thing is to motivate ourselves to change in a kind way, and not through blaming or criticizing ourselves about actions that are in the past.

Second, imagine that you have the strength to tolerate your difficulties and emotions, and the confidence to accept them without judging yourself.

Third, imagine you have a very warm and affectionate way of treating yourself. Imagine talking to yourself with warmth and kindness. Try to develop a kind facial expression while doing this, perhaps with a gentle smile.

Finally, commit to be there for yourself, to support yourself. Even in the most difficult situations, commit to not judging or blaming yourself for your mistakes. Instead, you help and give yourself what you need.

Keep breathing slowly. Offer yourself the following words of kindness and compassion and repeat them gently, feeling their importance:

- May you be happy (you might wish to use your name)
- May you be at peace
- May you be well
- May you be tranquil

Finally, take a few deep breaths and try to enjoy any feeling of calmness or tranquility that may arise. When you are ready, gently open your eyes.

**Relaxing Visualization 1 – Beach**

In this visualization exercise, we are going to create an image of ourselves doing something relaxing.

Place both feet on the ground, shoulder-width apart, and rest your hands on your legs. Close your eyes or look at the ground if you prefer.

Gently focus on your breath. Breathe from your abdomen a few times. Notice the flow of air going in and out through your nose. You don't need to change anything, just allow things to be as they are.

It's okay for your mind to wander – when this happens just notice it curiously and gently guide your mind back to the breath.

Feel your arms and legs letting go and relaxing… and your shoulders too…

When you're ready, imagine you're walking down a long stretch of white sand, toward the ocean… You're looking out at the water… it's blue and green.

You can hear the waves ahead… you can smell the sea breeze… you feel a nice cool breeze flowing…

The sand is hot, and very soft. Imagine taking off your shoes and walking on the beach.

Hear the waves crashing on the shore… Feel the clear smell of salt in the water.

Allow yourself to feel content and relaxed, allow these feelings to grow. Remember to keep your body posture as relaxed as possible.

Take a walk on the beach, by the edge of the water... release your worries... calm down... enjoy the moment...

Further on there is a comfortable chair with a towel, just for you…

Sit or lay down on the chair, or spread the towel on the sand… relax there… enjoy the sun… the breeze… the waves…

You feel calm and relaxed...

When you feel ready to return from that quiet place, slowly return to your usual level of alertness and awareness… Gently let the image fade, and leave the exercise, returning your attention to the room.

**Relaxing Visualization 2 – Forest**

In this visualization exercise we are going to create an image of ourselves doing something relaxing.

Place both feet flat on the ground, shoulder-width apart, and rest your hands on your legs. Close your eyes or look at the ground if you prefer.

Gently focus on your breath. Breathe from your abdomen a few times. Notice the flow of air going in and out through your nose. You don't need to change anything, just allow things to be as they are.

It's okay for your mind to wander – when this happens just notice it curiously and gently guide your mind back to the breath.

Feel your arms and legs letting go and relaxing… and your shoulders too…

When you're ready, imagine you're walking down a path through the woods. The trail is soft under your shoes, it's a mix of dirt, fallen leaves, and moss. As you walk, your body relaxes and your mind clears, more and more with each step.

Breathe in the fresh mountain air, fill your lungs completely. Now exhale, release all the air. Feel refreshed.

The air is cool, but pleasant. The sun filters through the trees, making a dappled pattern, moving on the ground in front of you.

Listen to the sounds of the forest… the birds sing. A gentle breeze blows. The leaves on the trees change and sway in the gentle wind.

As you walk through the woods, feel your muscles relax and lengthen. As your arms swing to the rhythm of your walk, they become loose, relaxed, and floppy.

Your legs and lower body also relax, they feel free and relaxed. Feel the tension leaving your body as you admire the scenery around you.

Ahead is a large, smooth rock, in the sun... like a chair waiting for you to rest. Sit or lie on the rock if you wish. It's very comfortable. You feel very comfortable and at ease. The sun shines on you. Enjoy this quiet place for a few moments.

When you are ready to leave this peaceful place, slowly begin to reawaken your body. Gently let the image fade, and leave the exercise, returning your attention to this room.
